# Supplementary figures and images for: Phytoplankton fatty acid proportions in the Canadian Arctic are strongly affected by temperature, salinity, and phosphate in late summer
Source: PLoS One. 2026 Jan 22;21(1):e0340414. doi: 10.1371/journal.pone.0340414 (PMC12826509; doi:10.1371/journal.pone.0340414)

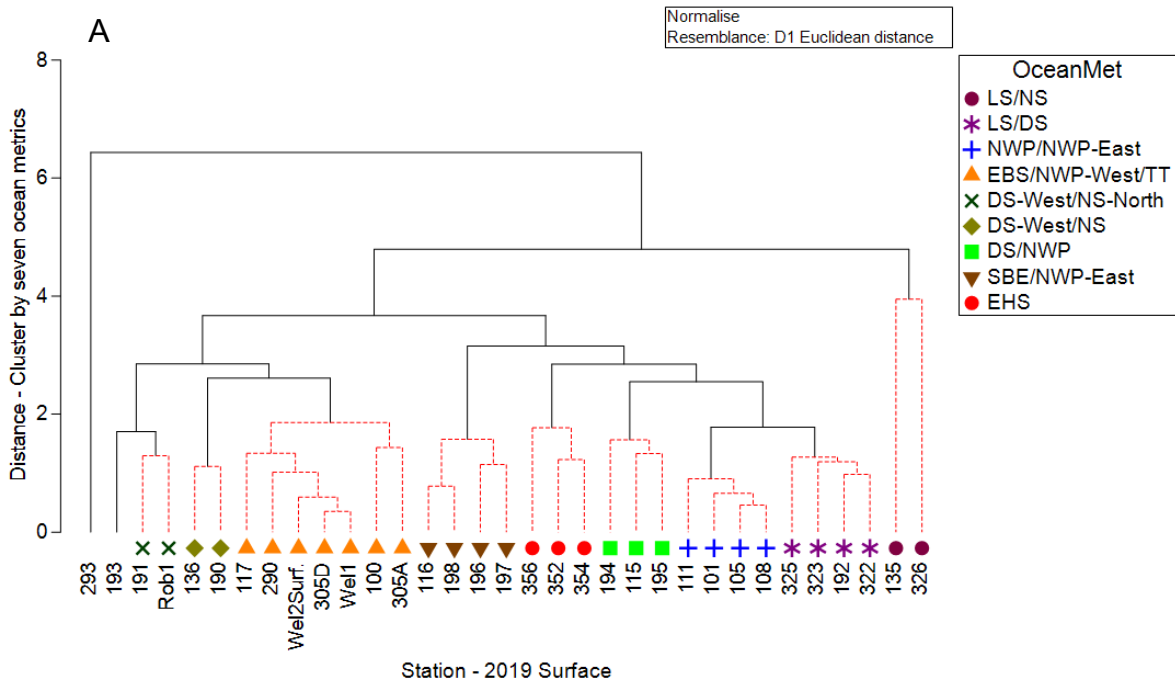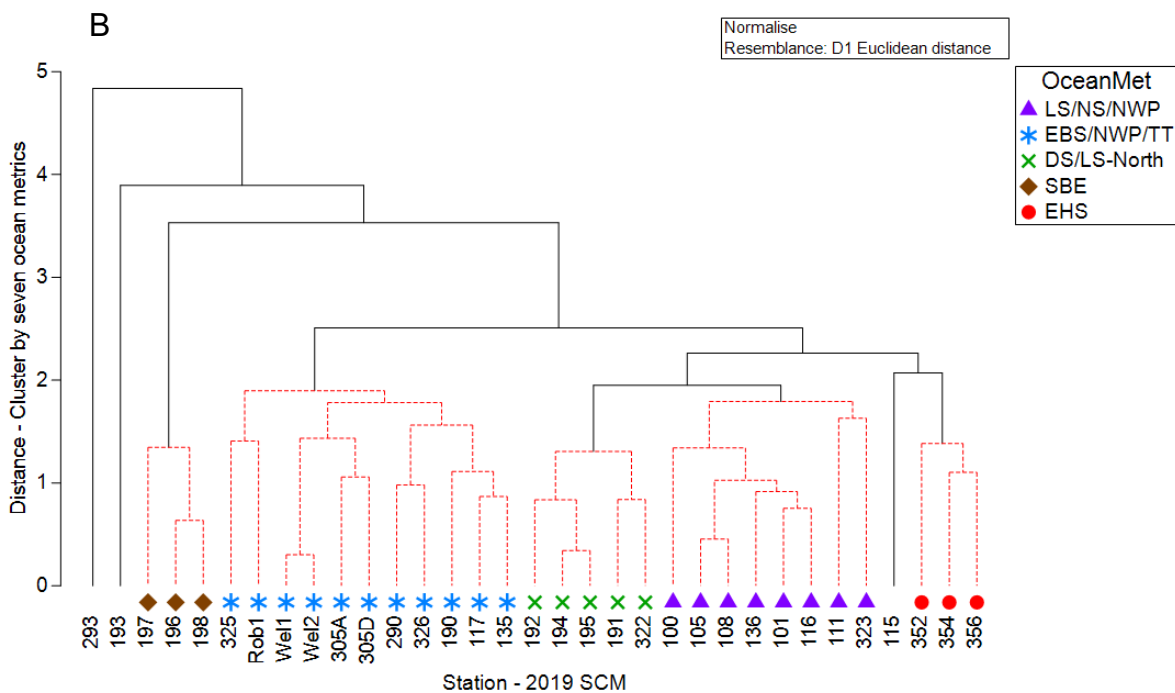

Supplement: S1 Fig — Hierarchical cluster analysis using group averages and similarity profile permutation tests (SIMPROF; red lines) based on seven oceanic metrics (bottom depth, temperature, salinity, light transmission, fluorescence, oxygen saturation, and dissolved oxygen) applied to samples gathered from surface waters (A) and the sub-surface chlorophyll maximum (SCM)(B) from July 8th – September 3rd, 2019. Station names and subsequent cluster grouping are termed OceanMet groups. Shorthand names include East Hudson Strait (EHS), Store Hellefiske Bank (SHB), North Water Polynya (NWP), Davis Strait (DS), Nares Strait (NS), Lancaster Sound (LS), East Barrow Strait (EBS), and Talbot Trough (TT). (PDF) [file pone.0340414.s002.pdf]

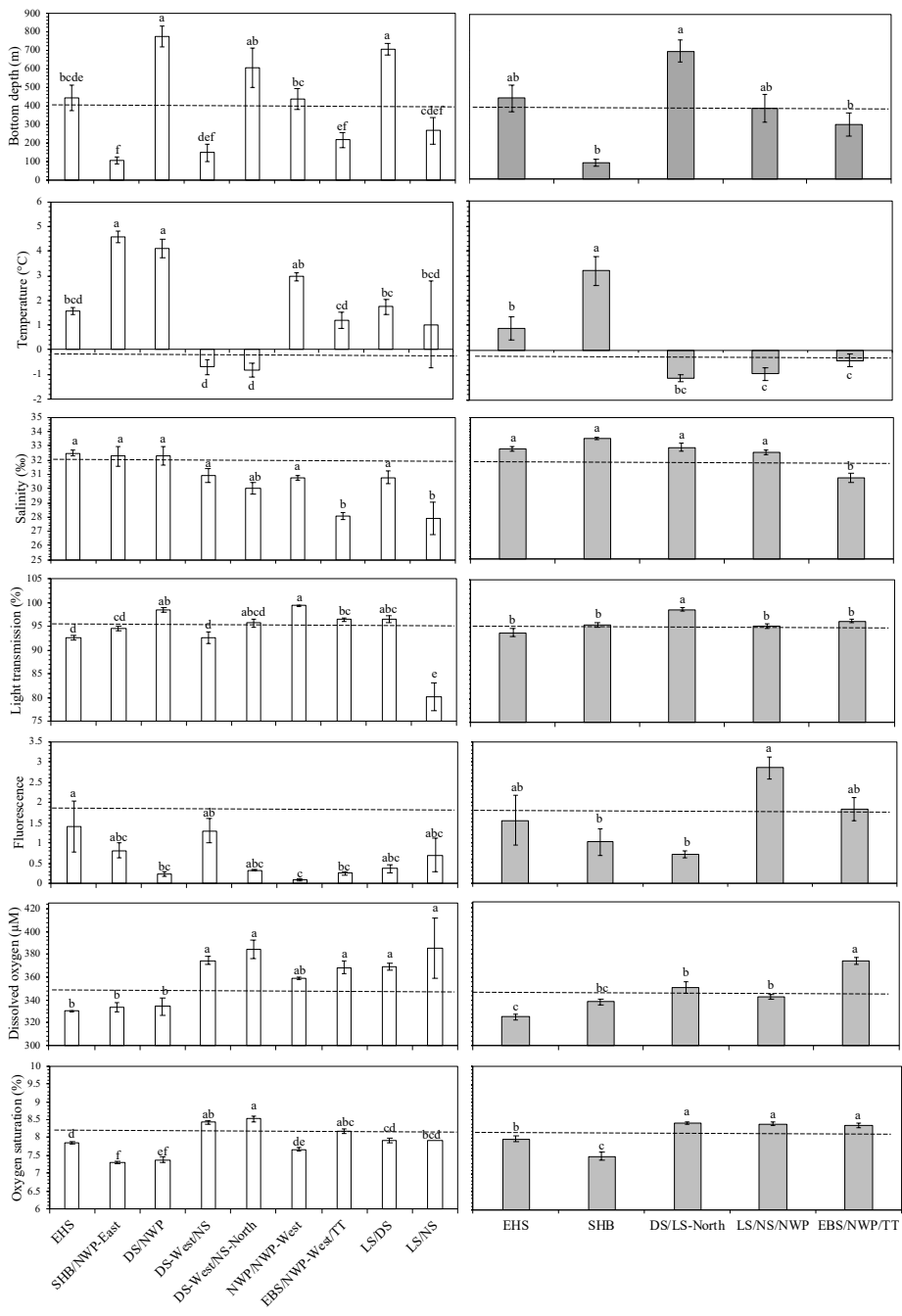

Supplement: S2 Fig — A summary of the seven ocean metrics averaged (±SE) across the OceanMet groups for both surface (white) and sub-surface chlorophyll maximum (grey) gathered from July 8th – September 3rd, 2019. Letters differentiate significantly different groups (ANOVA; Tukey, p < 0.5) and the horizontal bar represents the overall average of both surface and the sub-chlorophyll maximum. The OceanMet groups are named by location shorthand instead of ocean metric description; refer to Table 2 for shorthand ocean metric description which includes nutrient information in OceanMet group. Shorthand names include East Hudson Strait (EHS), Store Hellefiske Bank (SHB), North Water Polynya (NWP), Davis Strait (DS), Nares Strait (NS), Lancaster Sound (LS), East Barrow Strait (EBS), and Talbot Trough (TT). (PDF) [file pone.0340414.s003.pdf]

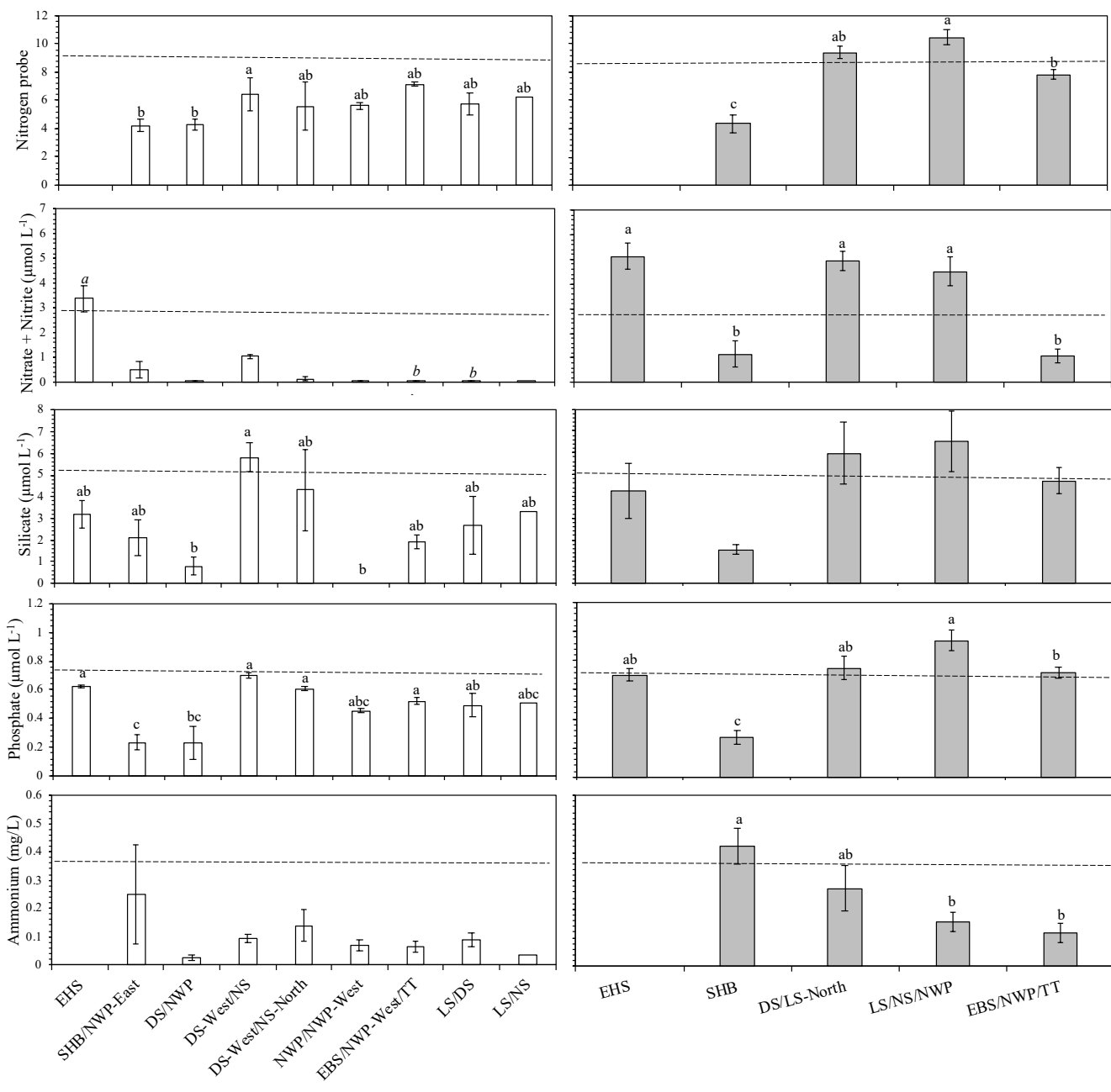

Supplement: S3 Fig — A summary of the six ocean nutrients averaged (±SE) across the OceanMet groups for both surface (white) and the sub-chlorophyll maximum (grey) gathered from July 8th – September 3rd, 2019. Letters differentiate significantly different groups (ANOVA; Tukey, p < 0.5) and the horizontal bar represents the overall average of both surface and the sub-surface chlorophyll maximum; a Kruskal Wallis test was performed for nitrate + nitrite surface, and the groups responsible for the difference are italicized. A blank column indicates variable was not collected. The OceanMet groups are named by location shorthand instead of ocean metric description; refer to Table 2 for shorthand ocean metric description which includes nutrient information in OceanMet group. Shorthand names include East Hudson Strait (EHS), Store Hellefiske Bank (SHB), North Water Polynya (NWP), Davis Strait (DS), Nares Strait (NS), Lancaster Sound (LS), East Barrow Strait (EBS), and Talbot Trough (TT). (PDF) [file pone.0340414.s004.pdf]

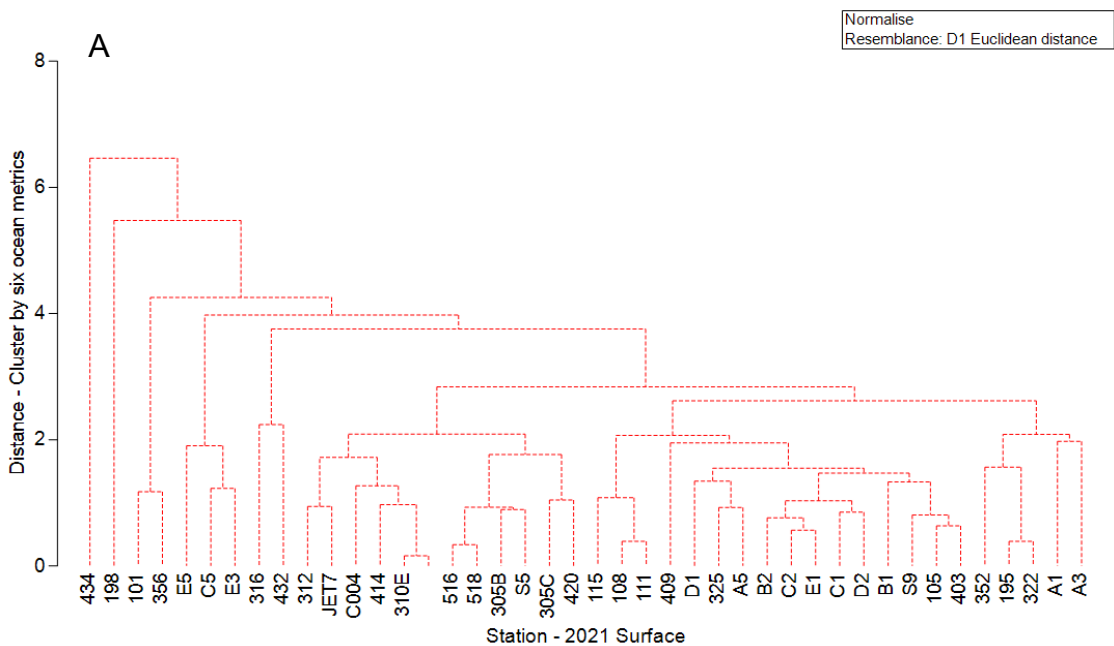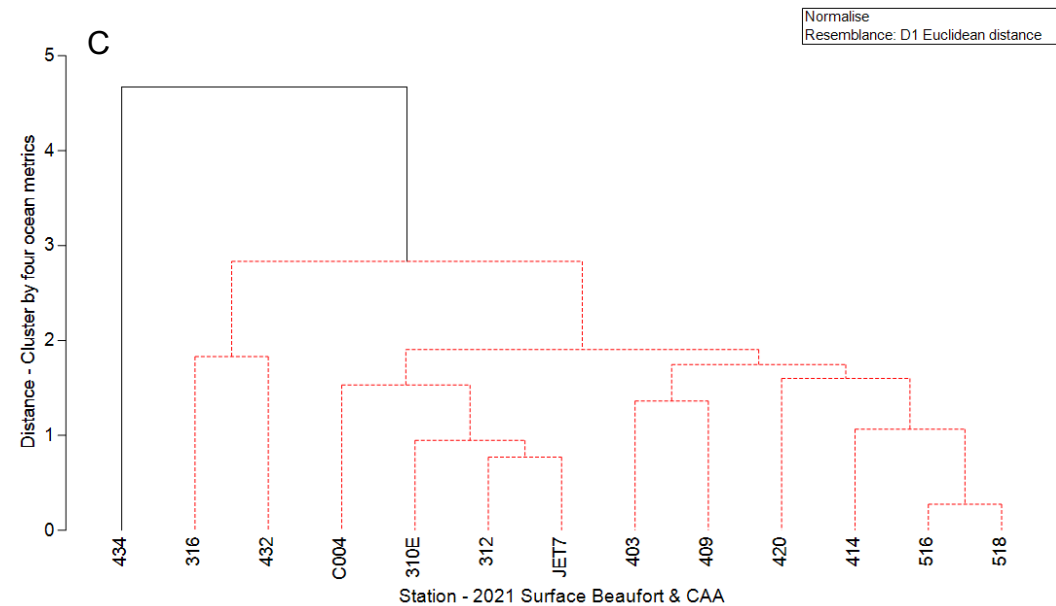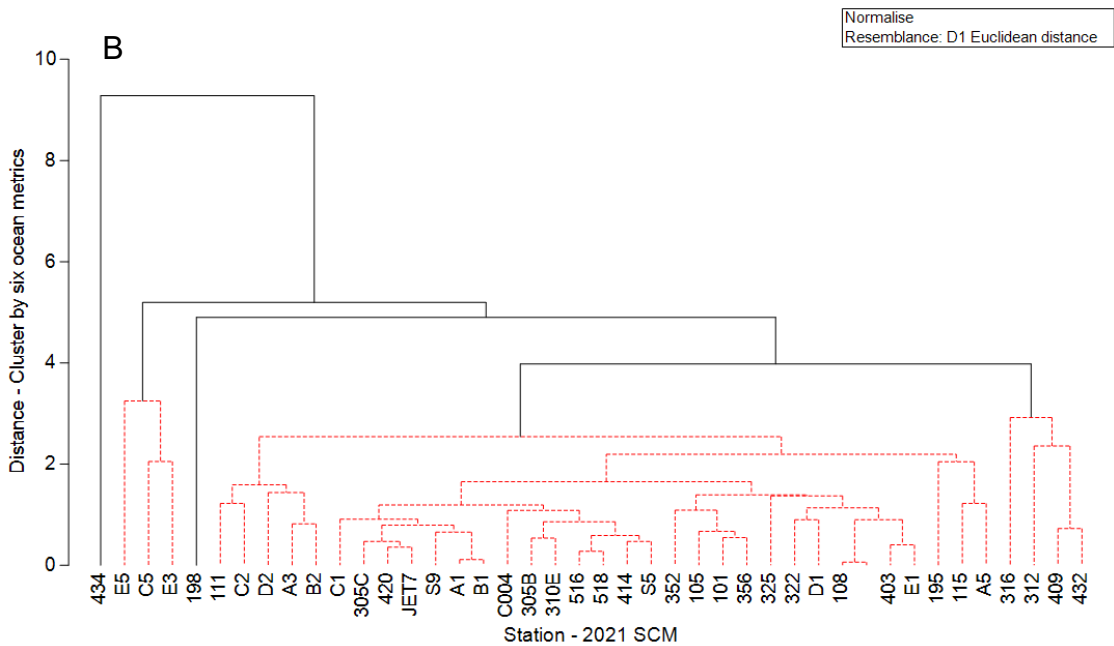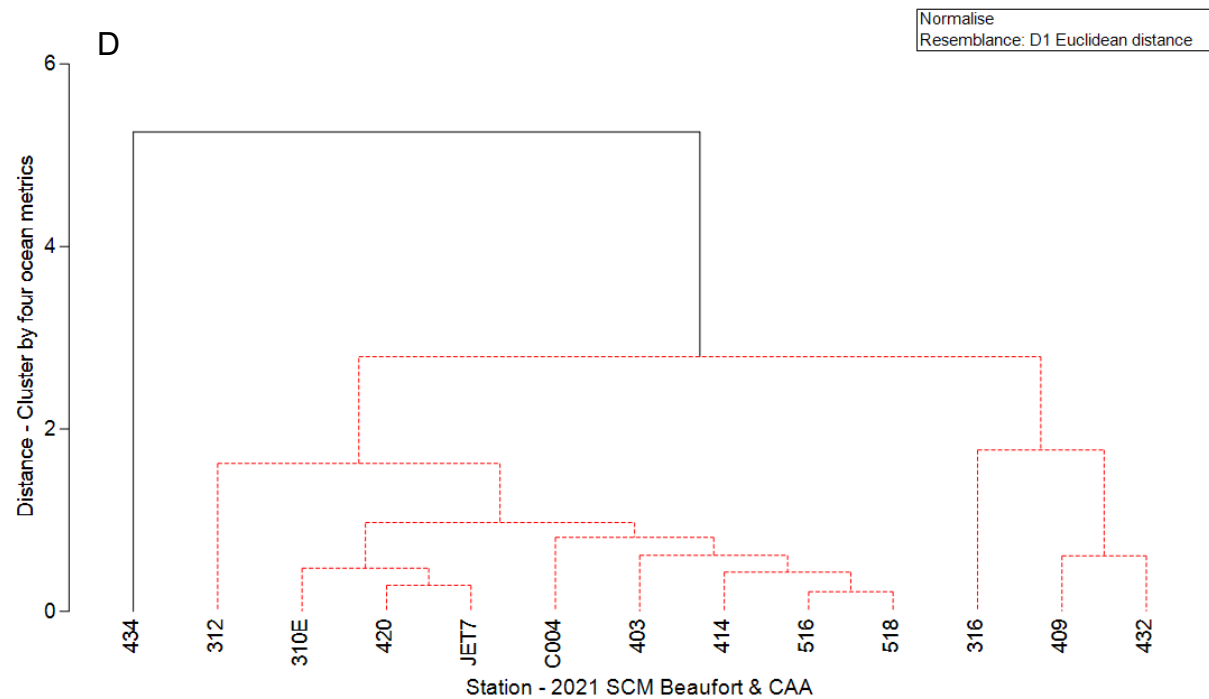

Supplement: S4 Fig — Hierarchical cluster analysis using group averages and similarity profile permutation tests (SIMPROF; red lines) based on six oceanic metrics (bottom depth, temperature, salinity, light transmission, fluorescence, and dissolved oxygen) applied to samples gathered from surface waters (A) and sub-surface chlorophyll maximum (SCM)(B) between August 15 – October 3, 2021. Non-significant clusters for both surface and SCM resulted in a division between Baffin Bay and Canadian Arctic Archipelago (CAA), and subsequent cluster analysis were done separately on each region. Based on four oceanic metrics (bottom depth, temperature, salinity, and fluorescence) clusters were not significant for CAA surface and SCM stations, resulting in a single OceanMet group. (PDF) [file pone.0340414.s005.pdf]

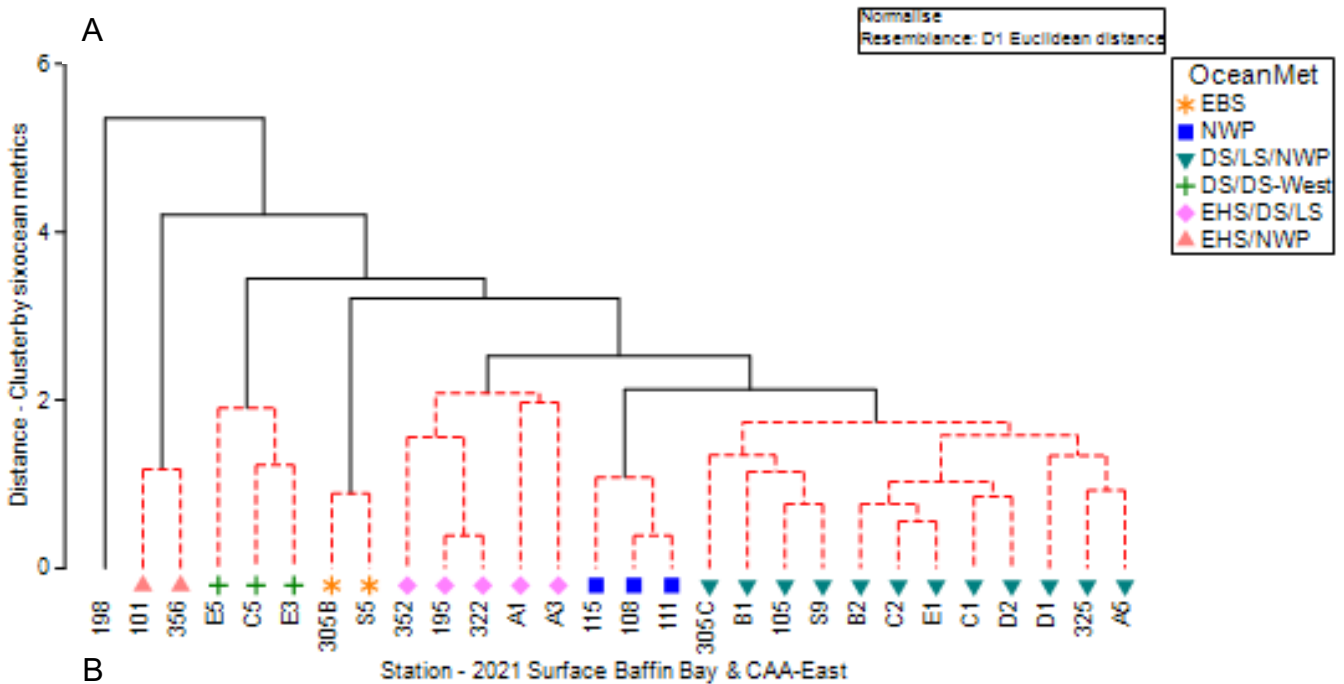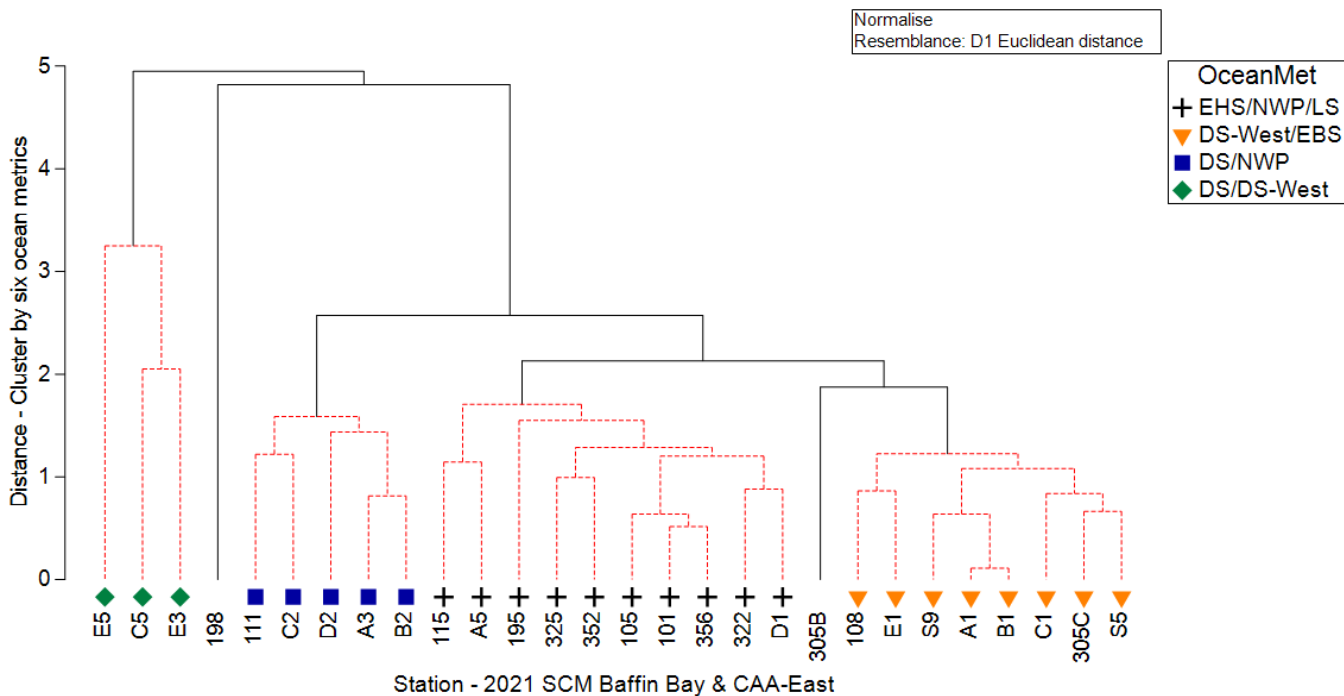

Supplement: S5 Fig — Hierarchical cluster analysis using group averages and similarity profile permutation tests (SIMPROF; red lines) based on six oceanic metrics (bottom depth, temperature, salinity, light transmission, fluorescence, and dissolved oxygen) applied to samples gathered from surface waters (A) and the sub-surface chlorophyll maximum (SCM)(B) from August 15 – October 3, 2021 in Baffin Bay and the east side of the Canadian Arctic Archipelago (CAA). Station names and subsequent cluster grouping are termed OceanMet groups. Shorthand names include East Hudson Strait (EHS), North Water Polynya (NWP), Davis Strait (DS), Lancaster Sound (LS), East Barrow Strait (EBS), Beaufort Sea (BF), and Canadian Arctic Archipelago (CAA). (PDF) [file pone.0340414.s006.pdf]

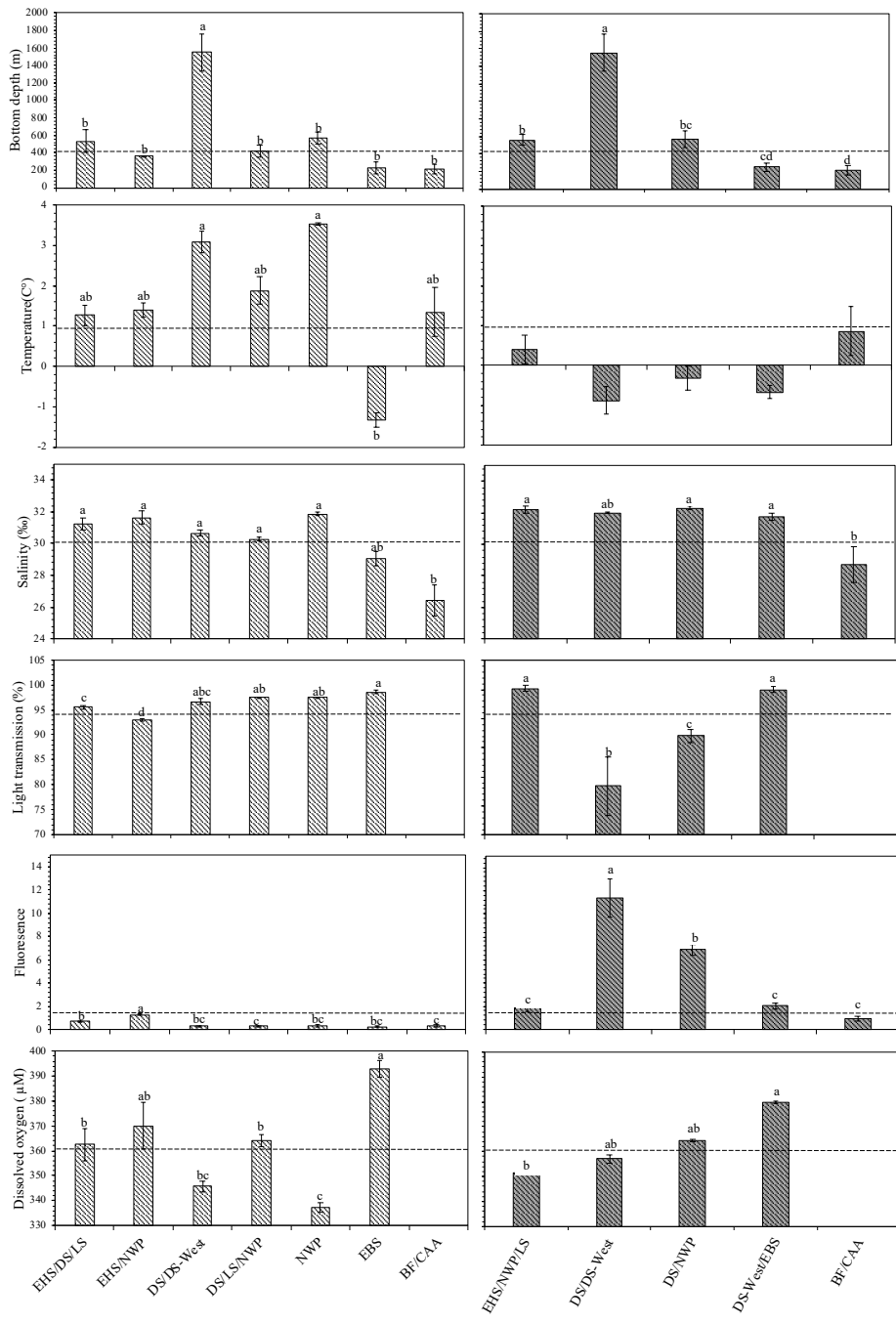

Supplement: S6 Fig — A summary of the six ocean metrics averaged (±SE) across the OceanMet groups for both surface (white striped) and sub-chlorophyll maximum (grey striped) gathered between August 15 – October 3, 2021. Letters differentiate significantly different groups (ANOVA; Tukey, p < 0.05) and the horizontal bar represents the overall average of both surface and sub-chlorophyll maximum. A blank column indicates variable was not collected. The OceanMet groups are named by location shorthand instead of ocean metric description; refer to Table 2 for shorthand ocean metric description which includes nutrient information in OceanMet group. Shorthand names include East Hudson Strait (EHS), North Water Polynya (NWP), Davis Strait (DS), Lancaster Sound (LS), East Barrow Strait (EBS), Beaufort Sea (BF), and Canadian Arctic Archipelago (CAA). (PDF) [file pone.0340414.s007.pdf]

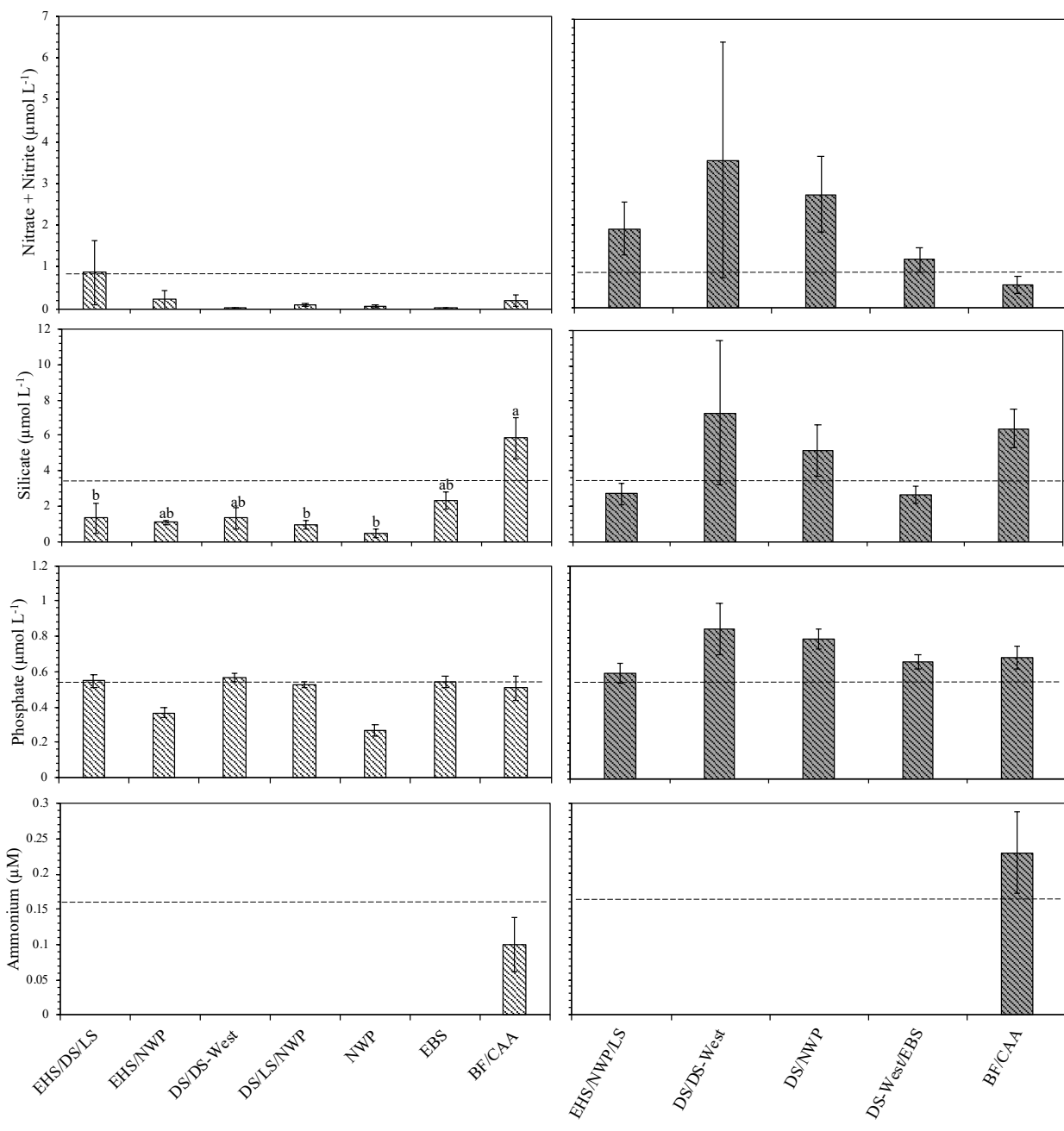

Supplement: S7 Fig — A summary of the five ocean nutrients averaged (±SE) across the OceanMet groups for both surface (white striped) and sub-surface chlorophyll maximum (grey striped) gathered from August 15 – October 3, 2021. Letters differentiate significantly different groups (ANOVA; Tukey, p < 0.05) and the horizontal bar represents the overall average of both surface and sub-surface chlorophyll maximum. A blank column indicates variable was not collected. The OceanMet groups are named by location shorthand instead of ocean metric description; refer to Table 2 for shorthand ocean metric description which includes nutrient information in OceanMet group. Shorthand names include East Hudson Strait (EHS), North Water Polynya (NWP), Davis Strait (DS), Lancaster Sound (LS), East Barrow Strait (EBS), Beaufort Sea (BF), and Canadian Arctic Archipelago (CAA). (PDF) [file pone.0340414.s008.pdf]
