# Supplementary material for: Phytoplankton fatty acid proportions in the Canadian Arctic are strongly affected by temperature, salinity, and phosphate in late summer
Source: PLoS One. 2026 Jan 22;21(1):e0340414. doi: 10.1371/journal.pone.0340414 (PMC12826509; doi:10.1371/journal.pone.0340414)
Supplement: S1 Table — Station locations from which both surface and sub-surface chlorophyll maximum (SCM) phytoplankton samples were collected in both 2019 and 2021. Stations are ordered by Arctic region and the most southern latitude. OceanMet groups (Figures S1-3) are also listed. Shorthand names include East Hudson Strait (EHS), Store Hellefiske Bank (SHB), North Water Polynya (NWP), Davis Strait (DS), Nares Strait (NS), Lancaster Sound (LS), East Barrow Strait (EBS), Talbot Trough (TT), Beaufort Sea (BF), and Canadian Arctic Archipelago (CAA). *The three filters were processed for lipids and fatty acids separately, and the values were later combined. (PDF) [file pone.0340414.s009.pdf]

Station	Date sampled	Arctic region	Arctic area	OceanMet Surface	OceanMet SCM	Filter *n*
356	3-Sep 2019; 15-Aug 2021	Hudson Bay System	East Hudson Strait	2019-EHS 2021-EHS/NWP	2019-EHS 2021-EHS/NWP/LS	1; 1
354	3-Sep 2019	Hudson Bay System	East Hudson Strait	2019-EHS	2019-EHS	1
352	3-Sep 2019; 16-Aug 2021	Hudson Bay System	East Hudson Strait	2019-EHS 2021-EHS/DS/LS	2019-EHS 2021-EHS/NWP/LS	1; 3
190	8-Jul 2019	Baffin Bay	Davis Strait-West	2019-DS-West/NS	2019-EBS/NWP/TT	1
A1	17-Aug 2021	Baffin Bay	Davis Strait-West	2021-EHS/DS/LS	2021-DS-West/EBS	3*
191	8-Jul 2019	Baffin Bay	Davis Strait-West	2019-DS-West/NS-North	2019-DS/LS-North	1
192	9-Jul 2019	Baffin Bay	Davis Strait	2019-LS/DS	2019-DS/LS-North	1
A3	18-Aug 2021	Baffin Bay	Davis Strait-West	2021-EHS/DS/LS	2021-DS/NWP	3*
193	9-Jul 2019	Baffin Bay	Davis Strait	Outlier	Outlier	1
194	10-Jul 2019	Baffin Bay	Davis Strait	2019-DS/NWP	2019-DS/LS-North	1
A5	19-Aug 2021	Baffin Bay	Davis Strait	2021-DS/LS/NWP	2021- EHS/NWP/LS	3*
195	10-Jul 2019; 19-Aug 2021	Baffin Bay	Davis Strait	2019-DS/NWP 2021-EHS/DS/LS	2019-DS/LS-North 2021-EHS/NWP/LS	1; 3
196	10-Jul 2019	Baffin Bay	Store Hellefiske Bank	2019-SHB/NWP-East	2019-SHB	1
197	10-Jul 2019	Baffin Bay	Store Hellefiske Bank	2019- SHB/NWP-East	2019-SHB	1
B1	22-Aug 2021	Baffin Bay	Davis Strait-West	2021-DS/LS/NWP	2021-DS-West/EBS	3*
198	10-Jul 2019; 20-Aug 2021	Baffin Bay	Store Hellefiske Bank-East	2019- SHB/NWP-East 2021-Outlier	2019-SHB 2021-Outlier	1; 3

B2	21-Aug 2021	Baffin Bay	Davis Strait-West	2021-DS/LS/NWP	2021-DS/NWP	3*
C1	23-Aug 2021	Baffin Bay	Davis Strait-West	2021- DS/LS/NWP	2021-DS-West/EBS	3*
D1	26-Aug 2021	Baffin Bay	Davis Strait-West	2021- DS/LS/NWP	2021-EHS/NWP/LS	3*
C2	23-Aug 2021	Baffin Bay	Davis Strait-West	2021- DS/LS/NWP	2021-DS/NWP	3*
D2	25-Aug 2021	Baffin Bay	Davis Strait-West	2021- DS/LS/NWP	2021-DS/NWP	3*
C5	24-Aug 2021	Baffin Bay	Davis Strait	2021-DS/DS-West	2021-DS/DS-West	3*
E1	27-Aug 2021	Baffin Bay	Davis Strait-West	2021- DS/LS/NWP	2021-DS-West/EBS	3*
E3	28-Aug 2021	Baffin Bay	Davis Strait-West	2021- DS/DS-West	2021-DS/DS-West	3*
E5	28-Aug 2021	Baffin Bay	Davis Strait	2021- DS/DS-West	2021-DS/DS-West	3*
293	7-Aug 2019	Baffin Bay	North Water Polynya-WestInlet	Outlier	Outlier	1
290	6-Aug 2019	Baffin Bay	North Water Polynya-WestInlet	2019-EBS/NWP-West/TT	2019-EBS/NWP/TT	1
108	21-Jul 2019;<br>1-Sep 2021	Baffin Bay	North Water Polynya	2019-NWP/NWP-East<br>2021-NWP	2019-LS/NS/NWP<br>2021-DS-West/EBS	1; 3
111	21-Jul 2019<br>1-Sep 2021	Baffin Bay	North Water Polynya	2019- NWP/NWP-East<br>2021-NWP	2019- LS/NS/NWP<br>2021-DS/NWP	1; 3
105	22-Jul 2019;<br>1-Sep 2021	Baffin Bay	North Water Polynya	2019- NWP/NWP-East<br>2021-EHS/NWP	2019- LS/NS/NWP<br>2021-EHS/NWP/LS	1; 3
115	20-Ju 2019l;<br>31-Aug 2021	Baffin Bay	North Water Polynya	2019-DS/NWP<br>2021-NWP	Outlier<br>2021-EHS/NWP/LS	1; 3

116	20-Jul 2019	Baffin Bay	North Water Polynya-East	2019-SHB/NWP-East	2019-LS/NS/NWP	1
101	23-Jul 2019; 2-Sep 2021	Baffin Bay	North Water Polynya-West	2019-NWP/NWP-East 2021-EHS/NWP	2019-LS/NS/NWP 2021-EHS/NWP/LS	1; 3
100	23-Jul 2019	Baffin Bay	North Water Polynya-West	2019-EBS/NWP-West/TT	2019-LS/NS/NWP	1
117	6-Aug 2019	Baffin Bay	Talbot Trough	2019- EBS/NWP-West/TT	2019-EBS/NWP/TT	1
136	31-Jul 2019	Baffin Bay	Nares Strait	2019-DS-West/NS	2019-LS/NS/NWP	1
135	31-Jul 2019	Baffin Bay	Nares Strait	2019-LS/NS	2019-EBS/NWP/TT	1
Rob1	2-Aug 2019	Baffin Bay	Nares Strait-North	2019-DS-West/NS-North	2019-EBS/NWP/TT	1
326	25-Jul 2019	Can. Arctic Archipel.	Lancaster Sound	2019-LS/NS	2019-EBS/NWP/TT	1
325	25-Jul 2019; 5-Sep 2021	Can. Arctic Archipel.	Lancaster Sound	2019-LS/DS 2021-DS/LS/NWP	2019-EBS/NWP/TT 2021-EHS/NWP/LS	1; 3
323	24-Jul 2019	Can. Arctic Archipel.	Lancaster Sound	2019-LS/DS	2019-LS/NS/NWP	1
S5	8-Sep 2021	Can. Arctic Archipel.	East Barrow Strait	2021-EBS	2021-DS-West/EBS	3*
305A	12-Aug 2019	Can. Arctic Archipel.	East Barrow Strait	2019-EBS/NWP-West/TT	2019-EBS/NWP/TT	1
305B	7-Sep 2021	Can. Arctic Archipel.	East Barrow Strait	2021-EBS	2021-Outlier	3*
S9	8-Sep 2021	Can. Arctic Archipel.	East Barrow Strait	2021-DS/LS/NWP	2021-DS-West/EBS	3*
305C	7-Sep 2021	Can. Arctic Archipel.	East Barrow Strait	2021-DS/LS/NWP	2021-DS-West/EBS	3*

322	24-Jul2019; 5-Sep 2021	Can. Arctic Archipel.	Lancaster Sound	2019-LS/DS 2021-EHS/DS/LS	2019-DS/LS-North 2021-EHS/NWP/LS	1; 3
305D	14-Aug 2019	Can. Arctic Archipel.	East Barrow Strait	2019-EBS/NWP-West/TT	2019-EBS/NWP/TT	1
Wel1	13-Aug 2019	Can. Arctic Archipel.	East Barrow Strait-North	2019-EBS/NWP-West/TT	2019-EBS/NWP/TT	1
Wel2	13-Aug 2019	Can. Arctic Archipel.	East Barrow Strait-North	2019-EBS/NWP-West/TT	2019-EBS/NWP/TT	1
316	15-Sep 2021	Can. Arctic Archipel.	Queen Maud Channel	2021-BF/CAA	2021-BF/CAA	3*
316	15-Sep 2021	Can. Arctic Archipel.	Queen Maud Channel	2021-BF/CAA	2021-BF/CAA	3*
312	13-Sep2021	Can. Arctic Archipel.	Queen Maud Channel	2021-BF/CAA	2021-BF/CAA	3*
403	17-Sep 2021	Can. Arctic Archipel.	Queen Maud Channel	2021-BF/CAA	2021-BF/CAA	2
310E	12-Sep 2021	Can. Arctic Archipel.	Prince of Wales Island	2021-BF/CAA	2021-BF/CAA	3*
414	19-Sep 2021	Can. Arctic Archipel.	CAA East	2021-BF/CAA	2021-BF/CAA	2
409	18-Sep 2021	Can. Arctic Archipel.	CAA East	2021-BF/CAA	2021-BF/CAA	2
C004	11-Sep 2021	Can. Arctic Archipel.	East Barrow Strait	2021-BF/CAA	2021-BF/CAA	2
420	19-Sep 2021	Beaufort Sea	Beaufort West	2021-BF/CAA	2021-BF/CAA	2
JET7	20-Sep 2021	Beaufort Sea	Beaufort West	2021-BF/CAA	2021-BF/CAA	2
518	4-Oct 2021	Beaufort Sea	Beaufort East	2021-BF/CAA	2021-BF/CAA	2
516	3-Oct 2021	Beaufort Sea	Beaufort East	2021-BF/CAA	2021-BF/CAA	2

514	3-Oct 2021	Beaufort Sea	Beaufort East	2021-BF/CAA	2021-BF/CAA	2
