# Supplementary material for: Phytoplankton fatty acid proportions in the Canadian Arctic are strongly affected by temperature, salinity, and phosphate in late summer
Source: PLoS One. 2026 Jan 22;21(1):e0340414. doi: 10.1371/journal.pone.0340414 (PMC12826509; doi:10.1371/journal.pone.0340414)
Supplement: S2 Table — Summary averages (±SE) of the nine phytoplankton OceanMet group lipid class percentages, lipid metrics, fatty acid percentages (>1%), and fatty acid biomarkers gathered from surface waters from July 8th – September 3rd, 2019. If significance (p ≤ 0.05) was found among groups based on a lipid or fatty acid, Tukey comparisons in the form of lowercase letters are displayed. Some fatty acids were not detected (ND). Shorthand names include East Hudson Strait (EHS), Store Hellefiske Bank (SHB), North Water Polynya (NWP), Davis Strait (DS), Nares Strait (NS), Lancaster Sound (LS), East Barrow Strait (EBS), and Talbot Trough (TT). (PDF) [file pone.0340414.s010.pdf]

Shorthand area name	EHS	SHB/ NWP-East	DS/NWP	DS-West/NS	DS-West/NS-North	NWP/ NWP-East	EBS/ NWP-West/TT	LS/DS	LS/NS
Shorthand ocean metric description	high nitrate	high temp/low oxygen sat.	high temp & depth/low oxygen sat.	high oxygen sat.	high oxygen sat. north	average	high oxygen sat./low salinity	high bottom depth (oceanic)	low salinity & light
Stations in group (*n*)	3	4	3	2	2	4	7	4	2
Total lipids (mg/g WW)	3.5 ± 0.7	5.6 ± 0.4	5.7 ± 1.3	9.6 ± 3.8	6.8 ± 1	7.7 ± 3.8	13 ± 9	6.9 ± 1.5	5.8 ± 2.6
HC%[1]	4 ± 1.4	0.2 ± 0.2	3.3 ± 0.9	6 ± 6	3.8 ± 1.2	5 ± 2.6	6.4 ± 2.7	6.8 ± 2.5	8.8 ± 5.1
TAG%[2]	13.4 ± 4	10.2 ± 1.8	14.7 ± 5.7	12.4 ± 1.2	19.6 ± 8.1	19.5 ± 6.6	22.2 ± 4	18.3 ± 4.7	33.2 ± 2.9
FFA%[3]	30.2 ± 1.4^{a}	2.03 ± 1.3^{b}	7.1 ± 5^{ab}	15 ± 5.9^{ab}	23 ± 11^{ab}	9.7 ± 5.3^{ab}	24.5 ± 3.5^{a}	8.8 ± 3.01^{ab}	10.5 ± 10.5^{ab}
ALC%[4]	9.3 ± 1.3	8.4 ± 2.4	8.1 ± 4.7	7.5 ± 3.4	8.8 ± 0.7	4.5 ± 1.5	6.7 ± 1.2	6.4 ± 3.2	3.3 ± 3.2
ST%[5]	2 ± 1	1.2 ± 0.4	1.4 ± 0.2	1.2 ± 0.1	1.5 ± 0.1	0.4 ± 0.2	2.7 ± 1	1.7 ± 0.5	2.2 ± 0.2
AMPL%[6]	21.1 ± 6.3	21.5 ± 4.7	21.1 ± 3.9	35.1 ± 10.8	22.5 ± 5.3	16.9 ± 3.5	15.3 ± 2.6	20.8 ± 5.3	29.1 ± 4.8
PL%[7]	18.4 ± 3.8	55.3 ± 8.4	42.7 ± 12.1	21.1 ± 7.9	19.4 ± 14.7	42.8 ± 12.5	17.2 ± 5	35 ± 14.5	10.8 ± 4.9
Polar lipids%[8]	39.6 ± 4.4^{ab}	76.9 ± 4.2^{a}	63.8 ± 13.5^{ab}	56.2 ± 18.7^{ab}	41.9 ± 20^{ab}	59.7 ± 9.2^{ab}	32.6 ± 4.7^{b}	55.8 ± 11.8^{ab}	39.9 ± 9.7^{ab}
TAG/PL	0.81 ± 0.3	0.22 ± 0.1	0.50 ± 0.3	0.71 ± 0.3	3.12 ± 2.8	0.70 ± 0.3	1.58 ± 0.6	1.84 ± 1.4	4.02 ± 2.1
TAG/ST	2 ± 1	1.2 ± 0.4	1.4 ± 0.2	1.2 ± 0.1	1.5 ± 0.1	0.4 ± 0.2	2.7 ± 1	1.7 ± 0.5	2.2 ± 0.2
14:0%	6.5 ± 0.4	8. ± 0.9	7.2 ± 0.5	10.5 ± 1.4	6.2 ± 0.2	6.5 ± 0.2	6.9 ± 0.4	7 ± 1.2	8.9 ± 1.2
16:0%	14.3 ± 1	15.5 ± 0.9	16.8 ± 0.7	18.4 ± 2.3	16.8 ± 2.1	15.5 ± 0.6	18.2 ± 2.1	17.6 ± 2.9	22.3 ± 1.9
18:0%	23.2 ± 8.1^{a}	6.4 ± 1.5^{ab}	5.4 ± 2.2^{ab}	8.7 ± 6.9^{ab}	6.3 ± 2.4^{ab}	11.1 ± 3.8^{ab}	8.6 ± 2.5^{ab}	4.4 ± 1.1^{b}	1.9 ± 1^{b}
20:0%	0.9 ± 0.3^{bc}	5.6 ± 0.9^{a}	5.3 ± 1.6^{ab}	1.3 ± 0.4^{abc}	4.9 ± 2.4^{abc}	1.7 ± 0.2^{abc}	0.5 ± 0.2^{c}	2 ± 1.1^{abc}	0.2 ± 0.2^{c}
ΣSFA%[9]	46.2 ± 8.8	36.6 ± 1.8	36 ± 1.5	39.9 ± 3.4	34.8 ± 2.8	35.7 ± 3.3	36.2 ± 2.6	31.9 ± 2.3	34 ± 0.8
16:1ω7%	10.9 ± 1.1^{bcd}	7.3 ± 0.7^{d}	9.3 ± 2.4^{cd}	22.4 ± 0.3^{abc}	15.9 ± 5^{abcd}	15 ± 0.9^{abcd}	21.2 ± 2.1^{ab}	16.8 ± 2.3^{abcd}	26.6 ± 8.3^{a}
16:4ω1%	1.7 ± 0.6^{ab}	0.3 ± 0.1^{b}	0.2 ± 0.2^{b}	2.5 ± 0.7^{a}	1.4 ± 0.8^{ab}	0.9 ± 0.1^{ab}	1.1 ± 0.3^{ab}	1.2 ± 0.4^{ab}	1.8 ± 0.4^{ab}
18:1ω9%	3.6 ± 16	4.1 ± 0.6	6.1 ± 2.1	3 ± 0.3	5.4 ± 0.1	3.5 ± 0.9	4 ± 1.2	3.9 ± 1	4 ± 1.6
18:1ω7%	1.7 ± 0.2	3.3 ± 0.5	3.8 ± 0.5	2.2 ± 0.01	2.4 ± 0.2	3.1 ± 0.1	1.7 ± 0.4	3.4 ± 1	2.3 ± 1.5
22:1ω9%	0.3 ± 0.1	ND	ND	0.7 ± 0.7	ND	0.5 ± 0.1	0.2 ± 0.1	0.3 ± 0.2	0.2 ± 0.2

ΣMUFA%[10]	19.7 ± 2.8^{c}	19.7 ± 0.8^{c}	22.5 ± 2.4^{bc}	30.6 ± 0.5^{abc}	25.9 ± 4.5^{abc}	24.± 1.9^{abc}	31.8 ± 1.8^{ab}	27.9 ± 2.6^{abc}	36.6 ± 4.3^{a}
16:3ω3%	0.5 ± 0.2	2.5 ± 1.3	0.7 ± 0.3	0.3 ± 0.1	0.1 ± 0.1	0.6 ± 0.2	1 ± 0.3	0.6 ± 0.2	0.1 ± 0.1
16:4ω3%	0.1 ± 0.1	1.9 ± 0.5	2.1 ± 0.7	0.3 ± 0.03	1.5 ±1	0.4 ± 0.1	0.6 ± 0.2	1.3 ± 0.7	0.9 ± 0.7
18:2ω6%	1.6 ± 0.7^{ab}	3.5 ± 0.7^{a}	3.2 ± 0.5^{ab}	2 ± 0.04^{ab}	3.9 ± 0.2^{ab}	1.9 ± 0.2^{ab}	1.5 ± 0.2^{b}	2.6 ± 0.6^{ab}	2.6 ± 1.3^{ab}
18:3ω3%	0.5 ± 0.2^{ab}	2.9 ± 0.5^{a}	3 ± 0.8^{ab}	0.8 ± 0.03^{ab}	2.0 ± 1.3^{ab}	1 ± 0.1^{ab}	0.9 ± 0.2^{b}	2.1 ± 0.6^{ab}	1.6 ± 1.2^{ab}
18:4ω3%	1.5 ± 0.5^{c}	6.3 ± 0.5^{a}	5.9 ± 1.4^{ab}	2.2 ± 0.1^{bc}	4.7 ± 1.7^{abc}	2.32± 0.02^{c}	2.2 ± 0.3^{c}	3.8 ± 1^{abc}	4 ± 1.2^{abc}
20:5ω3%	6.1 ± 1.4	7 ± 0.7	8.8 ± 1.2	8.5 ± 2.3	8.7 ± 1.7	14.4 ± 1.8	11.4 ± 1.8	12.1 ± 3.1	8.9 ± 1.3
22:5ω3%	8.7 ± 3.2	3.6 ± 0.9	2.7 ± 2.2	4.7 ± 1.2	5.6 ± 0.8	5.4 ± 0.8	4.5 ± 0.8	3.1 ± 0.5	0.5 ± 0.5
22:6ω3%	3.2 ± 1.3^{c}	8.1 ± 0.6^{abc}	10.4 ± 0.9^{a}	3.4 ± 1.3^{bc}	7 ± 0.3^{abc}	9.2 ± 0.5^{ab}	5.3 ± 1^{abc}	8.2 ± 1.8^{abc}	4.5 ± 1.4^{abc}
ΣPUFA%[11]	31.7 ± 6.7	39.2 ± 1.5	39 ± 0.7	27.1 ± 3.1	37.7 ± 1	38.3 ± 1.7	30.4 ± 2.4	37.1 ± 5.2	27.6 ± 2.9
PUFA/SFA	0.8 ± 0.3	1.1 ± 0.1	1.1 ± 0.03	0.7 ± 0.1	1.1 ± 0.1	1.1 ± 0.1	0.9 ± 0.1	1.2 ± 0.2	0.8 ± 0.1
Σω3%[12]	23.4 ± 5.7	32.3 ± 2	34.1 ± 0.1	20.2 ± 2.5	30.5 ± 3.2	33.7 ± 1.6	26.2 ± 2.5	31.4 ± 5.2	20.6 ± 2.4
Σω6%[13]	2.9 ± 0.5^{b}	7.1 ± 1.6^{a}	4.1 ± 0.3 ^{ab}	4.2 ± 0.1 ^{ab}	5.2 ± 1 ^{ab}	3.3 ± 0.4 ^{b}	2.7 ± 0.3 ^{b}	4.3 ± 0.53^{ab}	4.3 ± 0.7^{ab}
DHA/EPA[14]	0.5 ± 0.1^{b}	1.2 ± 0.1^{a}	1.24± 0.3^{a}	0.4 ± 0.1^{ab}	0.8 ± 0.2^{ab}	0.7 ± 0.1^{ab}	0.5 ± 0.1^{b}	0.7 ± 0.1^{ab}	0.5 ± 0.2^{b}
DHA + EPA%	9.3 ± 2.6	15.0 ± 1.2	19.2 ± 0.6	11.9 ± 3.6	15.7 ± 1.5	23.6 ± 2.3	16.7 ± 2.3	20.3 ± 4.7	13.4 ± 0.1
bacterial%[15]	3.6 ± 0.4^{ab}	7.2 ± 1.9^{a}	4.2 ± 0.2^{ab}	3.2± 0.1^{ab}	2.4 ± 0.9^{b}	2.9 ± 0.1^{b}	2.9 ± 0.3^{b}	4.3 ± 0.7^{ab}	2.1 ± 0.2^{b}
diatom[16]	0.8 ± 0.1^{ab}	0.5 ± 0.03^{b}	0.6 ± 0.2^{ab}	1.2 ± 0.2^{ab}	0.9 ± 0.2^{ab}	1 ± 0.04^{ab}	1.3 ± 0.2^{a}	1.02 ± 0.2^{ab}	1.2 ± 0.5^{ab}
flagellate[17]	0.6 ± 0.1	0.5 ± 0.3	0.3 ± 0.1	0.9 ± 0.1	0.4 ± 0.1	0.5 ± 0.1	0.8 ± 0.1	0.6 ± 0.2	0.5 ± 0.3
coastal margin%[18]	2.1 ± 0.9^{bc}	6.4 ± 0.9^{a}	6.3 ± 1.2^{ab}	2.8 ± 0.01^{abc}	6.6 ± 1.1^{abc}	3 ± 0.2^{abc}	2.3 ± 0.3^{c}	4.7 ± 1.2^{abc}	4.2 ± 2.4^{abc}

[1]Hydrocarbons
[2]Triacylglycerols
[3]Free fatty acids
[4]Alcohols
[5]Sterols
[6]Acetone mobile polar lipids
[7]Phospholipids
[8]Sum of AMPL and PL
[9]Saturated fatty acid (SFA) sum also includes 15:0, 17:0, 19:0, 20:0, 21:0, 22:0, 23:0, and 24:0
[10]Monounsaturated (MUFA) sum also includes 14:1, 15:1, 16:1ω11, 16:1ω9, 16:1ω5, 17:1, 18:1ω11, 18:1ω6, 18:1ω5, 22:1ω7, 24:1
[11]Polyunsaturated (PUFA) sum also includes 16:2ω4, 16:3ω4, 18:2, 18:2ω4, 18:3ω6, 18:3ω4, 18:3ω3, 18:4ω1, 18:5ω3, 20:2, 20:2ω6, 20:3ω6, 20:4ω6, 20:3ω3, 20:4ω3, 22:0, 22:2NMID, 22:2, 21:5ω3, 22:4ω6, 22:5ω6, and 22:4ω3

[12]Sum includes 18:3ω3, 18:5ω3, 20:3ω3, 20:4ω3, 21:5ω3, and 22:4ω3
[13]Sum includes 18:3ω6, 20:2ω6, 20:3ω6, 20:4ω6, 22:4ω6, and 22:5ω6
[14]Ratio is docosahexaenoic acid (DHA)/eicosapentaenoic acid (EPA)
[15]Bacterial biomarker sum includes *i*15:0, *ai*15:0, 15:0, 15:1, *i*16:0, *ai*16:0, *i*17:0, *ai*17:0, 17:0, 17:1, and 18:1ω6
[16]Diatom biomarker is the ratio 16:1ω7/16:0
[17]Flagellate ratio is C_{18}PUFA/C_{16}PUFA
[18]coastal margin biomarker is the sum of 18:3ω3 and 18:2ω6
