# Supplementary material for: Phytoplankton fatty acid proportions in the Canadian Arctic are strongly affected by temperature, salinity, and phosphate in late summer
Source: PLoS One. 2026 Jan 22;21(1):e0340414. doi: 10.1371/journal.pone.0340414 (PMC12826509; doi:10.1371/journal.pone.0340414)
Supplement: S3 Table — Permutational analysis of variance (PERMANOVA) pairwise results among the nine OceanMet groups (shorthand area name) created from phytoplankton gathered from surface waters from July 8th – September 3rd, 2019. Significance (p ≤ 0.05) between pairs is denoted by an asterisk next to the group. Shorthand names include East Hudson Strait (EHS), Store Hellefiske Bank (SHB), North Water Polynya (NWP), Davis Strait (DS), Nares Strait (NS), Lancaster Sound (LS), East Barrow Strait (EBS), and Talbot Trough (TT). (PDF) [file pone.0340414.s011.pdf]

Groups	t	p
EBS/NWP-West/TT, SHB/NWP-East	1.38	0.094
EBS/NWP-West/TT, DS/NWP*	1.72	0.024
EBS/NWP-West/TT, DS West/NS*	2.44	0.002
EBS/NWP-West/TT, LS/NS	0.93	0.615
EBS/NWP-West/TT, NWP/NWP-East	0.87	0.581
EBS/NWP-West/TT, DS-West/NS-North	1.29	0.130
EBS/NWP-West/TT, LS/DS	1.23	0.196
EBS/NWP-West/TT, EHS	0.80	0.712
SHB/NWP-East, DS/NWP*	2.17	0.027
SHB/NWP-East, DS West/NS*	2.78	0.022
SHB/NWP-East, LS/NS	2.15	0.083
SHB/NWP-East, NWP/NWP-East	1.76	0.067
SHB/NWP-East, DS-West/NS-North	1.83	0.065
SHB/NWP-East, LS/DS	1.39	0.051
SHB/NWP-East, EHS	1.42	0.085
DS/NWP, DS West/NS	1.05	0.382
DS/NWP, LS/NS	1.96	0.102
DS/NWP, NWP/NWP-East	2.47	0.191
DS/NWP, DS-West/NS-North	1.15	0.283
DS/NWP, LS/DS	0.73	0.705
DS/NWP, EHS	1.67	0.087
DS West/NS, LS/NS	2.41	0.067
DS West/NS, NWP/NWP-East	2.53	0.066
DS West/NS, DS-West/NS-North	1.45	0.070
DS West/NS, LS/DS	1.28	0.212
DS West/NS, EHS*	2.18	0.032
LS/NS, NWP/NWP-East	1.16	0.343
LS/NS, DS-West/NS-North	0.79	0.690
LS/NS, LS/DS	1.02	0.478
LS/NS, EHS	1.09	0.393
NWP/NWP-East, DS-West/NS-North	1.52	0.341
NWP/NWP-East, LS/DS	0.98	0.465
NWP/NWP-East, EHS	1.06	0.495
DS-West/NS-North, LS/DS	0.59	0.934
DS-West/NS-North, EHS	1.49	0.200
LS/DS, EHS	1.19	0.219
