# Supplementary material for: Phytoplankton fatty acid proportions in the Canadian Arctic are strongly affected by temperature, salinity, and phosphate in late summer
Source: PLoS One. 2026 Jan 22;21(1):e0340414. doi: 10.1371/journal.pone.0340414 (PMC12826509; doi:10.1371/journal.pone.0340414)
Supplement: S4 Table — Regression summary correlating the oceanic measurements (bottom depth, temperature, salinity, light transmission, dissolved oxygen, oxygen saturation, nitrate sensor, nitrate + nitrite, silicate, phosphate, and ammonium) with lipid class percentages, lipid biomarkers, fatty acid percentages, and fatty acid biomarkers gathered from surface (A) and subsurface chlorophyll maximum (SCM)(B) waters between July 10 – September 3, 2019 from the East Hudson Strait, through Baffin Bay, and ending in the east part of the Canadian Arctic Archipelago. Lipid and fatty acid classes presented are above, on average amongst all samples, 1% of the phytoplankton gathered from all locations in both 2019 and 2021. If a correlation was significant (p ≤ 0.05), the correlation coefficient was highlighted as follows: 1–0.5 = orange, 0.49–0 = yellow, −0.01 – −0.49 = light green, −0.5 – −1 = green. (PDF) [file pone.0340414.s012.pdf]

2019 Surface	Bottom Depth	Temp.	Salinity	Light Trans.	Fluores.	Diss. O_2	O_2 Satur.	Probe N_2	NO_3^-	SiO_4^-	PO_4^{3-}	NH_4^+
Total lipids (mg/g WW)	0.08	-0.11	-0.28	0.12	-0.09	0.12	0.17	0.14	-0.13	-0.01	0.01	-0.01
HC%[1]	0.04	-0.19	-0.42	-0.16	-0.11	0.32	0.27	0.36	-0.09	0.05	0.24	-0.24
TAG%[2]	-0.06	-0.19	-0.45	-0.12	-0.29	0.35	0.20	0.36	-0.26	-0.20	0.22	-0.30
FFA%[3]	-0.12	-0.43	-0.30	0.01	0.02	0.11	0.43	0.57	0.36	0.07	0.51	-0.17
ALC%[4]	-0.06	-0.01	0.20	0.22	-0.19	-0.12	-0.03	-0.28	0.19	0.03	-0.08	0.18
ST%[5]	-0.15	-0.05	-0.31	-0.04	-0.06	-0.02	0.10	0.22	0.03	0.15	0.18	-0.13
AMPL%[6]	0.12	-0.18	0.26	-0.35	0.26	0.10	0.09	-0.40	0.12	0.10	-0.08	-0.08
PL%[7]	0.09	0.46	0.39	0.20	0.10	-0.35	-0.46	-0.38	-0.14	-0.03	-0.42	0.31
Polar%[8]	0.14	0.39	0.51	0.04	0.22	-0.30	-0.42	-0.56	-0.09	0.01	-0.46	0.28
TAG/PL	0.07	-0.38	-0.35	-0.22	-0.12	0.52	0.34	0.37	-0.14	-0.07	0.26	-0.19
TAG/ST	-0.15	-0.05	-0.31	-0.04	-0.06	-0.02	0.10	0.22	0.03	0.15	0.18	-0.13
14:0%	-0.07	-0.12	0.09	-0.57	0.73	0.26	0.03	0.23	-0.10	0.02	0.01	-0.14
16:0%	-0.11	-0.11	-0.27	-0.30	0.01	0.33	0.12	0.16	-0.25	-0.05	-0.09	-0.21
18:0%	-0.19	0.01	0.17	0.13	-0.04	-0.40	-0.02	-0.08	0.67	-0.04	0.23	0.06
20:0%	0.26	0.29	0.41	0.27	-0.17	-0.30	-0.30	-0.66	-0.07	0.22	-0.42	0.49
∑SFA%[9]	-0.22	0.03	0.22	-0.08	0.14	-0.31	-0.06	-0.17	0.56	0.01	0.04	0.08
16:1ω7%	-0.16	-0.62	-0.59	-0.35	0.18	0.77	0.65	0.75	-0.26	0.01	0.51	-0.32
18:1ω9%	0.11	0.14	0.02	0.14	-0.23	-0.08	-0.10	-0.25	-0.15	0.04	-0.27	0.12
18:1ω7%	0.21	0.46	0.41	0.27	-0.31	-0.32	-0.46	-0.71	-0.25	-0.21	-0.62	0.03
22:1ω9%	0.04	-0.22	-0.06	-0.21	0.41	0.32	0.20	0.50	0.07	-0.02	0.34	-0.12
∑MUFA%[10]	-0.18	-0.49	-0.60	-0.34	0.12	0.73	0.52	0.67	-0.40	-0.03	0.29	-0.33
16:3ω3%	-0.33	0.36	0.11	0.09	-0.02	-0.32	-0.33	-0.04	-0.11	-0.12	-0.20	-0.09
16:4ω3%	0.34	0.17	0.17	-0.15	0.30	-0.06	-0.14	-0.30	-0.26	0.22	-0.29	0.19
16:4ω1%	-0.11	-0.52	-0.26	-0.24	0.03	0.35	0.48	0.55	0.33	0.38	0.65	-0.09
18:2ω6%	0.19	0.27	0.34	0.06	-0.17	-0.18	-0.27	-0.67	-0.24	0.13	-0.50	0.30
18:3ω3%	0.30	0.29	0.25	0.14	-0.23	-0.22	-0.26	-0.68	-0.23	0.27	-0.45	0.44
18:4ω3%	0.23	0.34	0.28	0.06	-0.16	-0.22	-0.34	-0.67	-0.26	0.22	-0.51	0.38
20:5ω3%	0.19	-0.03	-0.34	0.30	-0.30	0.26	0.10	0.38	-0.39	-0.24	0.15	-0.16
22:5ω3%	-0.15	-0.13	0.14	0.24	-0.02	-0.23	0.14	0.02	0.36	0.16	0.35	0.11
22:6ω3%	0.43	0.45	0.20	0.44	-0.42	-0.27	-0.41	-0.40	-0.45	-0.23	-0.41	0.09
∑PUFA%[11]	0.38	0.32	0.24	0.37	-0.25	-0.29	-0.29	-0.34	-0.19	0.02	-0.20	0.20
PUFA/SFA	0.38	0.16	0.05	0.30	-0.27	-0.05	-0.11	-0.10	-0.31	0.06	-0.06	0.08
∑ω3%[12]	0.36	0.34	0.15	0.45	-0.35	-0.25	-0.28	-0.32	-0.34	-0.04	-0.23	0.19
∑ω6%[13]	-0.06	0.25	0.32	-0.11	0.10	-0.19	-0.27	-0.34	-0.15	0.12	-0.32	0.14
DHA/EPA[14]	0.25	0.55	0.45	0.24	-0.23	-0.48	-0.55	-0.76	-0.21	-0.08	-0.68	0.25
DHA% + EPA%	0.35	0.21	-0.13	0.42	-0.41	0.04	-0.14	0.06	-0.49	-0.27	-0.11	-0.07
Bacterial%[15]	-0.16	0.49	0.45	0.08	0.07	-0.47	-0.51	-0.41	0.02	-0.08	-0.43	0.03
Diatom[16]	-0.11	-0.60	-0.54	-0.17	0.14	0.65	-0.28	-0.72	-0.16	0.22	-0.50	0.40
Flagellate[17]	-0.27	-0.26	-0.27	-0.25	0.41	0.29	0.61	0.68	-0.02	-0.01	0.55	-0.25
Coastal margin%[18]	0.26	0.30	0.31	0.11	-0.21	-0.21	0.27	0.62	-0.24	0.06	0.38	-0.25

2019 SCM	Bottom Depth	Temp.	Salinity	Light Trans.	Fluores.	Diss. O_2	O_2 Satur.	Probe N_2	NO_3^-	SiO_4^-	PO_4^{3-}	NH_4^+
Total lipids (mg/g WW)	0.06	0.05	0.34	-0.27	0.45	-0.25	-0.10	0.05	0.15	0.12	0.11	0.17
HC%[1]	-0.14	-0.20	-0.13	0.27	-0.17	0.17	0.22	0.17	-0.01	0.14	0.17	-0.04
TAG%[2]	0.04	-0.17	-0.21	0.14	-0.16	0.02	0.18	0.22	0.12	0.09	0.16	-0.20
FFA%[3]	0.07	0.12	-0.09	-0.04	-0.09	-0.03	-0.11	0.04	0.00	-0.31	-0.15	0.09
ALC%[4]	-0.09	0.21	0.36	0.12	-0.34	-0.33	-0.26	-0.22	0.07	-0.37	-0.36	0.58
ST%[5]	0.02	-0.14	-0.02	0.12	-0.03	0.24	0.14	0.06	-0.15	-0.09	0.03	0.05
AMPL%[6]	-0.11	-0.07	-0.13	0.11	0.05	0.11	0.04	-0.14	-0.11	-0.20	-0.18	-0.25
PL%[7]	0.13	0.03	0.26	-0.22	0.26	-0.13	-0.05	-0.01	0.11	0.29	0.16	0.08
Polar%[8]	0.10	0.00	0.25	-0.21	0.34	-0.09	-0.04	-0.08	0.07	0.24	0.10	-0.02
TAG/PL	-0.16	0.10	-0.54	0.05	-0.23	0.19	0.00	-0.07	-0.24	-0.37	-0.24	-0.23
TAG/ST	0.02	-0.14	-0.02	0.12	-0.03	0.24	0.14	0.06	-0.15	-0.09	0.03	0.05
14:0%	-0.09	0.13	0.04	-0.70	0.60	0.02	-0.14	-0.08	-0.05	-0.11	-0.06	-0.15
16:0%	0.20	-0.11	-0.26	-0.23	0.19	0.17	0.15	0.06	-0.08	0.02	0.07	-0.44
18:0%	-0.08	0.08	-0.08	0.08	-0.05	0.01	-0.09	0.04	-0.02	-0.12	-0.09	0.13
20:0%	0.08	0.23	0.35	0.27	-0.40	-0.12	-0.29	-0.36	-0.03	-0.12	-0.42	0.33
∑SFA%[9]	-0.02	0.16	-0.06	-0.09	0.04	0.03	-0.18	-0.07	-0.07	-0.19	-0.22	0.06
16:1ω7%	0.03	-0.29	-0.27	-0.37	0.52	0.14	0.35	0.31	0.02	0.12	0.39	-0.41
18:1ω9%	0.05	-0.09	0.11	0.31	-0.26	-0.07	-0.01	-0.07	0.11	0.23	-0.04	-0.02
18:1ω7%	0.14	0.31	0.47	0.35	-0.49	-0.23	-0.37	-0.34	0.00	-0.25	-0.53	0.44
22:1ω9%	0.28	-0.26	-0.18	-0.47	0.59	0.20	0.29	0.26	0.00	0.02	0.35	-0.21
∑MUFA%[10]	0.05	-0.24	-0.28	-0.33	0.45	0.21	0.30	0.15	-0.07	0.12	0.30	-0.38
16:3ω3%	0.13	-0.16	0.09	0.31	-0.31	-0.32	0.16	0.24	0.26	0.38	0.16	-0.08
16:4ω3%	0.13	0.13	-0.04	-0.17	-0.10	0.27	-0.11	-0.37	-0.29	-0.16	-0.33	-0.01
16:4ω1%	-0.13	-0.29	-0.25	0.06	0.22	0.11	0.30	0.32	0.09	0.30	0.45	-0.32
18:2ω6%	0.10	0.08	0.45	0.20	-0.28	-0.18	-0.18	-0.24	0.13	-0.13	-0.34	0.46
18:3ω3%	-0.01	0.27	0.28	0.22	-0.40	-0.10	-0.31	-0.42	-0.08	-0.04	-0.39	0.25
18:4ω3%	0.18	0.22	0.25	0.35	-0.53	-0.25	-0.25	-0.21	0.06	0.04	-0.32	0.11
20:5ω3%	-0.01	-0.37	-0.13	0.03	0.19	0.12	0.42	0.36	0.07	0.18	0.53	-0.14
22:5ω3%	-0.20	0.20	0.38	0.25	-0.34	-0.32	-0.29	-0.20	0.19	-0.05	-0.25	0.51
22:6ω3%	0.17	0.18	0.26	0.26	-0.46	-0.03	-0.19	-0.32	-0.06	-0.07	-0.26	0.24
∑PUFA%[11]	-0.01	-0.02	0.28	0.38	-0.40	-0.19	-0.01	-0.03	0.14	0.13	0.05	0.26
PUFA/SFA	-0.06	-0.15	0.10	0.27	-0.22	-0.03	0.16	0.07	0.05	0.20	0.24	0.07
∑ω3%[12]	0.05	-0.05	0.27	0.40	-0.46	-0.20	0.03	0.00	0.15	0.16	0.07	0.23
∑ω6%[13]	-0.27	0.28	0.24	-0.07	0.02	-0.02	-0.31	-0.49	-0.17	-0.24	-0.40	0.27
DHA/EPA[14]	0.07	0.42	0.32	0.19	-0.50	-0.17	-0.46	-0.45	-0.08	-0.24	-0.55	0.29
DHA% + EPA%	0.05	-0.29	-0.03	0.11	0.03	0.10	0.33	0.24	0.05	0.14	0.42	-0.05
Bacterial%[15]	-0.09	0.08	0.11	0.13	-0.20	-0.14	-0.07	-0.26	-0.02	0.05	-0.19	-0.04
Diatom[16]	-0.08	-0.32	-0.16	-0.24	0.45	0.06	0.36	0.33	0.09	0.16	0.45	-0.21
Flagellate[17]	-0.13	-0.28	-0.30	-0.14	0.30	0.12	0.35	0.31	0.02	0.21	0.42	-0.31
Coastal margin%[18]	0.05	0.19	0.40	0.23	-0.37	-0.15	-0.27	-0.36	0.03	-0.09	-0.40	0.39
