# Supplementary material for: Phytoplankton fatty acid proportions in the Canadian Arctic are strongly affected by temperature, salinity, and phosphate in late summer
Source: PLoS One. 2026 Jan 22;21(1):e0340414. doi: 10.1371/journal.pone.0340414 (PMC12826509; doi:10.1371/journal.pone.0340414)
Supplement: S6 Table — Permutational analysis of variance (PERMANOVA) pairwise results among the nine OceanMet groups (shorthand area name) created from phytoplankton gathered from sub-surface chlorophyll maximum (SCM) waters from July 8th – September 3rd, 2019. Significance (p ≤ 0.05) between pairs is denoted by an asterisk next to the group. Shorthand names include East Hudson Strait (EHS), Store Hellefiske Bank (SHB), North Water Polynya (NWP), Davis Strait (DS), Nares Strait (NS), Lancaster Sound (LS), East Barrow Strait (EBS), and Talbot Trough (TT). (PDF) [file pone.0340414.s014.pdf]

Groups	t	p
LS/NS/NWP, EBS/NWP/TT*	1.86	0.010
LS/NS/NWP, DS/LS-North*	2.74	0.001
LS/NS/NWP, SBE*	3.09	0.008
LS/NS/NWP, EHS*	2.28	0.008
EBS/NWP/TT, DS/LS-North	1.35	0.123
EBS/NWP/TT, SBE*	2.10	0.005
EBS/NWP/TT, EHS	0.66	0.849
DS/LS-North, SBE	1.61	0.081
DS/LS-North, EHS	1.32	0.187
SBE, EHS	2.49	0.105
