# Supplementary material for: Phytoplankton fatty acid proportions in the Canadian Arctic are strongly affected by temperature, salinity, and phosphate in late summer
Source: PLoS One. 2026 Jan 22;21(1):e0340414. doi: 10.1371/journal.pone.0340414 (PMC12826509; doi:10.1371/journal.pone.0340414)
Supplement: S7 Table — Summary averages (±SE) of the seven phytoplankton OceanMet group lipid class percentages, lipid metrics, fatty acid percentages (>1%), and fatty acid biomarkers gathered from surface waters from August 15 – October 4, 2021. If significance (p ≤ 0.05) was found among groups based on a lipid or fatty acid (Table S1), Tukey comparisons in the form of lowercase letters are displayed. Some fatty acids and lipids were not detected (ND). Shorthand names include East Hudson Strait (EHS), North Water Polynya (NWP), Davis Strait (DS), Lancaster Sound (LS), East Barrow Strait (EBS), Beaufort Sea (BF), and Canadian Arctic Archipelago (CAA). (PDF) [file pone.0340414.s015.pdf]

Shorthand area name	EHS/NWP	EHS/DS/LS	DS/DS-West	DS/LS/NWP	NWP	EBS	BF/CAA
Shorthand ocean metric description	low light/high fluorescence	average temperature	large bottom depth (oceanic)	average temperature and light transmission	low oxygen	high oxygen/low temp	high silica/low salinity
Stations in group (*n*)	2	5	3	12	3	2	14
Total lipids (mg/g WW)	35.9 ± 30.2	24.9 ± 11.2	23.9 ± 7	20.4 ± 4.9	9.4 ± 1.3	17 ± 1.4	16.3 ± 3.5
HC%[1]	4.1 ± 3.4	4.4 ± 1.5	4.7 ± 1.4	4.5 ± 0.8	11.1 ± 6.2	7.8 ± 1.9	3.3 ± 0.7
TAG%[2]	15.2 ± 15.2	1.1 ± 1.1	3 ± 1.5	8.6 ± 3.3	17.5 ± 8.9	ND	10.7 ± 3.3
FFA%[3]	18.7 ± 14.4	13.1 ± 3.9	18.7 ± 8.7	10.9 ± 3	23.9 ± 11.7	16.4 ± 1	7.9 ± 2.3
ALC%[4]	0.1 ± 0.1	ND	0.2 ± 0.2	0.3 ± 0.2	0.3 ± 0.3	0.8 ± 0.8	0.3 ± 0.1
ST%[5]	ND	0.2 ± 0.2[b]	0.5 ± 0.5[b]	0.01 ± 0.01[b]	0.5 ± 0.2[b]	1.7 ± 0.5[a]	0.02 ± 0.02[b]
AMPL%[6]	12.8 ± 10.5	14.7 ± 7	12.6 ± 5.9	10.7 ± 2.4	14.3 ± 3.5	14.4 ± 5.6	12.3 ± 2.7
PL%[7]	49 ± 43.3	66.4 ± 12.2	60.2 ± 13.9	64.7 ± 6.5	31.7 ± 11.4	58.9 ± 4.3	63.9 ± 5.9
Polar lipids%[8]	61.8 ± 32.8	81.1 ± 5.6	72.8 ± 9.1	75.4 ± 4.8	46 ± 8.8	73.3 ± 1.2	76.2 ± 3.7
TAG/PL	2.7 ± 2.7[a]	0.04 ± 0.04[b]	0.04 ± 0.02[b]	0.2 ± 0.1[b]	0.5 ± 0.3[b]		0.3 ± 0.1[b]
TAG/ST		0.2 ± 0.2	0.5 ± 0.5	0.01 ± 0.01	0.5 ± 0.2		0.02 ± 0.02
14:0%	11.5 ± 1.7[a]	8.6 ± 1.6[a]	9.9 ± 0.8[a]	8.2 ± 0.6[a]	10.1 ± 1.2[a]	6.8 ± 3.1[ab]	3.5 ± 0.5[b]
16:0%	19.7 ± 1.2[ab]	17.8 ± 1.5[ab]	17.2 ± 1.8[ab]	15.9 ± 0.5[a]	19.2 ± 0.9[ab]	18.8 ± 0.3[ab]	23.8 ± 1.9[b]
18:0%	13.7 ± 8.2[ab]	26.6 ± 9.2[ab]	7.7 ± 2.7[b]	14.1 ± 3.5[b]	14.2 ± 3.4[b]	11.6 ± 5.8[b]	39.5 ± 2.1[a]
20:0%	1 ± 0.5	1.4 ± 0.4	1.9 ± 0.3	3.1 ± 0.6	3 ± 1.2	1.4 ± 0.2	1.2 ± 0.1
ΣSFA%[9]	46.7 ± 8.4[ab]	54.9 ± 8.5[ab]	36.2 ± 3[b]	42 ± 3.5[b]	46.8 ± 4[b]	40.6 ± 4.1[b]	72.3 ± 2.4[a]
16:1ω7%	19.1 ± 0.2[a]	10.1 ± 3.2[ab]	18.3 ± 4.1[a]	10.7 ± 1[a]	9.5 ± 0.8[ab]	12.3 ± 6[ab]	4.6 ± 1.1[b]
16:4ω1%	1 ± 0.1[ab]	1.4 ± 0.2[a]	1.4 ± 0.3[a]	1 ± 0.2 [a]	0.8 ± 0.2[ab]	0.8 ± 0.4[ab]	0.3 ± 0.1[b]
18:1ω9%	6 ± 2.3	3.8 ± 0.7	3 ± 0.5	4.5 ± 1.1	7.3 ± 2.6	3.9 ± 0.2	3.8 ± 0.3
18:1ω7%	1.3 ± 0.2[ab]	1.3 ± 0.2[ab]	1.5 ± 0.2[ab]	1.6 ± 0.1[a]	1.3 ± 0.2[ab]	1.8 ± 0.7[ab]	1.1 ± 0.1[b]
22:1ω9%	228 ± 1.2[ab]	2.5 ± 1.5[b]	3.32± 0.7[ab]	4.5 ± 0.9[ab]	6.8 ± 2.6[ab]	13.30± 11.7[a]	0.1 ± 0.04[b]

ΣMUFA%[10]	34.3 ± 5.1[b]	22.7 ± 5.3 [ab]	31.8 ± 2.[b]	29.1 ± 2[b]	32.3 ± 3.2[b]	37.2 ± 3.6[b]	13.2 ± 1.4[a]
16:3ω3%	0.7 ± 0.1[ab]	0.7 ± 0.1[a]	0.8 ± 0.1[a]	0.5 ± 0.1[ab]	0.6 ± 0.1[ab]	0.6 ± 0.3[ab]	0.2 ± 0.1[b]
16:4ω3%	0.30± 0.1	0.8 ± 0.2	1.2 ± 0.4	1.7 ± 0.3	0.6 ± 0.3	0.8 ± 0.3	1.4 ± 0.2
18:2ω6%	2 ± 0.7	1.2 ± 0.3	1.9 ± 0.2	1.5 ± 0.1	1.6 ± 0.4	1.7 ± 0.4	1.8 ± 0.2
18:3ω3%	0.5 ± 0.02	0.7 ± 0.2	1 ± 0.3	1.3 ± 0.2	1.1 ± 0.5	1 ± 0.2	1.3 ± 0.2
18:4ω3%	1.6 ± 0.01	2.1 ± 0.6	2.8 ± 0.4	3.04 ± 0.4	2 ± 0.5	2.1 ± 0.6	2.2 ± 0.3
20:5ω3%	5.5 ± 1.8[abc]	8 ± 1.8[ab]	13.4 ± 3[a]	8.6 ± 0.9[ab]	4.8 ± 0.8[bc]	6.4 ± 2.7[abc]	2.1 ± 0.4[c]
22:5ω3%	0.6 ± 0.6[ab]	0.7 ± 0.3[b]	0.3 ± 0.2[ab]	2.5 ± 0.6[ab]	2.2 ± 1.2[ab]	2 ± 1.8[ab]	4.4 ± 0.7[a]
22:6ω3%	4.3 ± 2[ab]	4.1 ± 0.6[ab]	6.1 ± 0.5[a]	6.4 ± 0.6[a]	6 ± 1.5[a]	5.4 ± 2.2[ab]	1.5 ± 0.2[b]
ΣPUFA%[11]	17.3 ± 3.5[ab]	20.9 ± 3.3[ab]	30.4 ± 2.1[a]	26.9 ± 2.2[a]	19.1 ± 2.7[ab]	20.8 ± 7.1[ab]	13.4 ± 1.3[b]
PUFA/SFA	0.4 ± 0.1 [ab]	0.5 ± 0.1[ab]	0.9 ± 0.1[a]	0.7 ± 0.1[a]	0.4 ± 0.1[ab]	0.5 ± 0.2[ab]	0.2 ± 0.03[b]
Σω3%[12]	12.6 ± 3.8[ab]	16.4 ± 2.8[ab]	25.3 ± 1.7[a]	22.8 ± 2[a]	14.8 ± 2.7[ab]	16.7 ± 5.5[ab]	10.3 ± 1.1[b]
Σω6%[13]	3.6 ± 0.6	2.7 ± 0.6	3.6 ± 0.2	2.8 ± 0.2	3.1 ± 0.5	3 ± 1.2	2.6 ± 0.2
DHA/EPA[14]	0.7 ± 0.1	0.5 ± 0.1	0.5 ± 0.2	0.8 ± 0.1	1.3 ± 0.3	0.9 ± 0.01	0.9 ± 0.1
DHA + EPA%	9.8 ± 3.8	12.1 ± 2.3	19.5 ± 2.5	15.0 ± 1.3	10.7 ± 1.9	11.8 ± 4.8	3.6 ± 0.5
bacterial%[15]	3.2 ± 0.03[ab]	3 ± 0.5[ab]	2.9 ± 0.3[ab]	3.5 ± 0.2[a]	3 ± 0.1[ab]	2.9 ± 1.3[ab]	2.1 ± 0.2[b]
diatom[16]	1 ± 0.1[a]	0.6 ± 0.2[ab]	1.1 ± 0.3[a]	0.7 ± 0.1[a]	0.5 ± 0.03[ab]	0.7 ± 0.3[ab]	0.2 ± 0.1[b]
flagellate[17]	0.5 ± 0.2	0.8 ± 0.1	0.6 ± 0.1	0.6 ± 0.1	0.5 ± 0.2	0.5 ± 0.1	0.4 ± 0.1
coastal margin%[18]	2.6 ± 0.7	1.9 ± 0.4	2.9 ± 0.4	2.8 ± 0.2	2.8 ± 1	2.7 ± 0.6	3.1 ± 0.4

[1]Hydrocarbons
[2]Triacylglycerols
[3]Free fatty acids
[4]Alcohols
[5]Sterols
[6]Acetone mobile polar lipids
[7]Phospholipids
[8]Sum of AMPL and PL
[9]Saturated fatty acid (SFA) sum also includes 15:0, 17:0, 19:0, 20:0, 21:0, 22:0, 23:0, and 24:0
[10]Monounsaturated (MUFA) sum also includes 14:1, 15:1, 16:1ω11, 16:1ω9, 16:1ω5, 17:1, 18:1ω11, 18:1ω6, 18:1ω5, 22:1ω7, 24:1

[11]Polyunsaturated (PUFA) sum also includes 16:2ω4, 16:3ω4, 18:2, 18:2ω4, 18:3ω6, 18:3ω4, 18:3ω3, 18:4ω1, 18:5ω3, 20:2, 20:2ω6, 20:3ω6, 20:4ω6, 20:3ω3, 20:4ω3, 22:0, 22:2NMID, 22:2, 21:5ω3, 22:4ω6, 22:5ω6, and 22:4ω3

[12]Sum includes 18:3ω3, 18:5ω3, 20:3ω3, 20:4ω3, 21:5ω3, and 22:4ω3

[13]Sum includes 18:3ω6, 20:2ω6, 20:3ω6, 20:4ω6, 22:4ω6, and 22:5ω6

[14]Ratio is docosahexaenoic acid (DHA)/eicosapentaenoic acid (EPA)

[15]Bacterial biomarker sum includes *i*15:0, *ai*15:0, 15:0, 15:1, *i*16:0, *ai*16:0, *i*17:0, *ai*17:0, 17:0, 17:1, and 18:1ω6

[16]Diatom biomarker is the ratio 16:1ω7/16:0

[17]Flagellate ratio is C_{18}PUFA/C_{16}PUFA

[18]coastal margin biomarker is the sum of 18:3ω3 and 18:2ω6
