# Supplementary material for: Phytoplankton fatty acid proportions in the Canadian Arctic are strongly affected by temperature, salinity, and phosphate in late summer
Source: PLoS One. 2026 Jan 22;21(1):e0340414. doi: 10.1371/journal.pone.0340414 (PMC12826509; doi:10.1371/journal.pone.0340414)
Supplement: S8 Table — Permutational analysis of variance (PERMANOVA) pairwise results among the nine OceanMet groups (shorthand area name) created from phytoplankton gathered from surface waters from August 15 – October 4, 2021. Significance (p ≤ 0.05) between pairs is denoted by an asterisk next to the group. (PDF) [file pone.0340414.s016.pdf]

Groups	t	p
EHS/NWP, DS/LS/NWP	0.96	0.378
EHS/NWP, NWP	0.84	0.610
EHS/NWP, EHS/DS/LS	0.88	0.669
EHS/NWP, EBS	1.01	0.697
EHS/NWP, BF/CAA	1.48	0.089
EHS/NWP, DS/DS-West	1.02	0.363
DS/LS/NWP, NWP	1.37	0.110
DS/LS/NWP, EHS/DS/LS	1.10	0.301
DS/LS/NWP, EBS	1.56	0.117
DS/LS/NWP, BF/CAA*	2.99	0.001
DS/LS/NWP, DS/DS-West	1.12	0.223
NWP, EHS/DS/LS	1.11	0.255
NWP, EBS	1.09	0.418
NWP, BF/CAA	1.51	0.076
NWP, DS/DS-West	1.57	0.101
EHS/DS/LS, EBS	0.99	0.579
EHS/DS/LS, BF/CAA*	1.85	0.008
EHS/DS/LS, DS/DS-West	1.13	0.320
EBS, BF/CAA	1.65	0.059
EBS, DS/DS-West	1.32	0.214
BF/CAA, DS/DS-West*	2.33	0.003
