# Supplementary material for: Phytoplankton fatty acid proportions in the Canadian Arctic are strongly affected by temperature, salinity, and phosphate in late summer
Source: PLoS One. 2026 Jan 22;21(1):e0340414. doi: 10.1371/journal.pone.0340414 (PMC12826509; doi:10.1371/journal.pone.0340414)
Supplement: S9 Table — Regression summary correlating the oceanic measurements (bottom depth, temperature, salinity, light transmission, dissolved oxygen, oxygen saturation, nitrate sensor, nitrate + nitrite, silicate, phosphate, and ammonium) with lipid class percentages, lipid biomarkers, fatty acid percentages, and fatty acid biomarkers gathered from surface (A) and sub-surface chlorophyll maximum (SCM)(B) waters between August 15 – October 4, 2021 from the East Hudson Strait, through Baffin Bay and the Canadian Arctic Archipelago, ending in the Beaufort Sea. Lipid and fatty acid classes presented are above, on average amongst all samples, 1% of the phytoplankton gathered from all locations in both 2019 and 2021. If a correlation was significant (p ≤ 0.05), the correlation coefficient was highlighted as follows: 1–0.5 = orange, 0.49–0 = yellow, −0.01 – −0.49 = light green, −0.5 – −1 = green. *Ammonium was only taken from the Beaufort Sea and Canadian Arctic Archipelago stations, so results only reflect correlation with those stations. ^Light transmission and dissolved oxygen were only taken from the East Hudson Strait, Baffin Bay, and the eastern Canadian Arctic Archipelago stations, so results only reflect correlations with those stations. (PDF) [file pone.0340414.s017.pdf]

2021 Surface	Bottom Depth	Temp.	Salinity	Light Trans.	Fluores	Diss. O_2	NO_3^-	SiO_4^-	PO_4^{3-}	NH_4^+
Total lipids (mg/g WW)	0.14	-0.10	0.21	-0.16	0.15	0.03	0.00	-0.19	0.06	-0.30
HC%[1]	-0.11	0.28	-0.15	0.05	-0.05	-0.11	-0.06	-0.24	-0.15	0.41
TAG%[2]	0.29	0.02	0.30	-0.09	0.06	-0.02	-0.15	0.09	-0.09	0.61
FFA%[3]	-0.21	0.09	-0.01	0.05	-0.10	-0.10	-0.07	-0.22	-0.23	-0.15
ALC%[4]	0.14	-0.18	0.11	0.26	-0.07	0.22	0.07	0.03	-0.04	0.43
ST%[5]	0.13	0.18	-0.11	-0.08	-0.05	0.00	-0.12	-0.15	-0.04	-0.26
AMPL%[6]	-0.15	-0.22	-0.04	0.01	0.06	0.05	-0.14	-0.60	-0.06	0.62
PL%[7]	-0.14	-0.21	-0.11	-0.03	0.05	0.07	0.17	0.11	0.22	-0.60
Polar%[8]	-0.14	-0.21	-0.11	-0.03	0.05	0.07	0.17	0.12	0.27	-0.55
TAG/PL	-0.08	0.06	0.03	-0.33	0.32	0.15	-0.11	-0.06	-0.15	0.77
TAG/ST	0.14	-0.18	0.11	0.26	-0.07	0.22	-0.12	-0.15	-0.04	-0.26
14:0%	0.45	0.10	0.59	-0.24	0.24	0.10	-0.20	-0.59	-0.21	-0.30
16:0%	-0.11	-0.33	-0.10	-0.02	-0.08	-0.13	0.15	0.27	0.01	-0.28
18:0%	-0.39	-0.16	-0.47	-0.06	-0.06	-0.03	0.25	0.52	0.23	0.42
20:0%	-0.03	0.41	0.14	-0.06	0.09	-0.36	0.02	-0.11	-0.19	0.38
∑SFA%[9]	-0.37	-0.23	-0.42	-0.07	-0.07	-0.05	0.24	0.51	0.20	-0.18
16:1ω7%	0.54	-0.01	0.39	-0.21	0.18	0.19	-0.18	-0.46	-0.08	-0.15
18:1ω9%	-0.01	-0.04	0.18	0.05	-0.02	0.06	-0.16	-0.20	-0.31	-0.21
18:1ω7%	0.08	0.12	0.20	0.17	0.03	0.04	-0.29	-0.32	-0.20	-0.11
22:1ω9%	0.11	-0.12	0.25	0.39	-0.17	0.30	-0.11	-0.27	-0.05	0.15
∑MUFA%[10]	0.37	0.03	0.48	0.12	0.06	0.26	-0.11	-0.54	-0.22	-0.06
16:3ω3%	0.41	-0.06	0.42	-0.24	0.20	0.19	-0.14	-0.48	-0.06	0.22
16:4ω3%	-0.15	0.37	-0.31	0.22	-0.26	-0.23	-0.11	0.14	0.04	0.78
16:4ω1%	0.43	-0.04	0.44	-0.16	0.17	0.25	-0.07	-0.46	0.09	0.12
18:2ω6%	-0.07	0.15	-0.01	-0.18	0.06	-0.35	-0.09	0.08	-0.21	-0.20
18:3ω3%	-0.26	0.55	-0.23	-0.15	0.18	-0.50	-0.05	0.27	-0.42	-0.16
18:4ω3%	-0.06	0.51	-0.06	-0.15	0.19	-0.39	-0.05	0.08	-0.28	0.00
20:5ω3%	0.55	0.20	0.36	-0.04	0.02	0.08	-0.21	-0.47	0.07	0.47
22:5ω3%	-0.40	-0.08	-0.40	0.29	-0.29	-0.08	0.00	0.38	0.16	-0.01
22:6ω3%	0.19	0.28	0.46	0.12	0.09	-0.10	-0.17	0.35	-0.25	-0.23
∑PUFA%[11]	0.28	0.40	0.26	-0.02	0.07	-0.17	-0.20	-0.34	-0.13	0.32
PUFA/SFA	0.37	0.32	0.36	0.00	0.05	-0.15	-0.19	-0.42	-0.09	0.27
∑ω3%[12]	0.26	0.42	0.24	0.01	0.05	-0.18	-0.20	-0.32	-0.12	0.36
∑ω6%[13]	0.17	0.19	0.09	-0.16	0.19	-0.17	-0.21	-0.09	-0.28	-0.08
DHA/EPA[14]	-0.28	0.16	0.01	0.11	0.05	-0.27	-0.02	0.18	-0.43	-0.49
DHA% + EPA%	0.45	0.25	0.44	0.02	0.05	0.42	-0.21	-0.52	-0.06	0.28
Bacterial%[15]	0.15	0.34	0.33	-0.01	0.16	-0.20	-0.08	-0.41	-0.19	0.58
Diatom[16]	0.54	0.06	0.36	-0.15	0.12	0.18	-0.22	-0.46	-0.04	-0.05
Flagellate[17]	0.27	-0.08	0.20	-0.12	0.06	0.27	0.04	-0.30	0.18	0.76
Coastal margin%[18]	-0.20	0.43	-0.16	-0.17	0.15	-0.49	-0.08	0.21	-0.37	-0.20

2021 SCM	Bottom Depth	Temp.	Salinity	Light Trans.	Fluores.	Diss. O_2	NO_3^-	SiO_4^-	PO_4^{3-}	NH_4^+
Total lipids (mg/g WW)	-0.11	0.02	-0.07	-0.05	-0.04	0.02	-0.11	0.04	0.00	0.11
HC%[1]	0.01	-0.09	0.12	0.13	-0.13	0.00	0.07	-0.11	0.10	0.42
TAG%[2]	0.12	-0.17	0.16	-0.05	0.03	0.04	-0.02	-0.13	0.12	0.05
FFA%[3]	0.55	-0.18	0.27	-0.55	0.58	-0.03	0.25	0.02	0.16	0.15
ALC%[4]	0.03	-0.12	0.07	0.25	-0.18	0.21	-0.06	-0.05	0.07	-0.03
ST%[5]	0.29	-0.12	0.12	-0.47	0.15	-0.11	-0.16	-0.13	0.00	-0.47
AMPL%[6]	0.29	-0.23	0.14	-0.26	0.25	0.18	0.18	0.00	0.18	0.08
PL%[7]	-0.45	0.29	-0.30	0.38	-0.36	-0.10	-0.20	0.08	-0.23	-0.16
Polar%[8]	-0.42	0.25	-0.31	0.33	-0.32	-0.01	-0.16	0.11	-0.20	-0.18
TAG/PL	0.11	-0.09	0.05	0.10	-0.07	0.05	0.05	-0.04	0.08	0.05
TAG/ST	0.29	-0.12	0.12	-0.47	0.15	-0.11	-0.16	-0.13	0.00	-0.47
14:0%	0.54	-0.21	0.46	-0.66	0.72	0.03	0.22	-0.28	-0.05	-0.22
16:0%	-0.31	0.10	-0.08	0.49	-0.44	-0.18	-0.19	-0.10	-0.08	-0.05
18:0%	-0.52	0.18	-0.47	0.68	-0.60	-0.01	-0.25	0.28	0.08	0.53
20:0%	-0.04	0.13	0.12	0.07	0.01	0.03	-0.07	-0.21	-0.19	-0.21
∑SFA%[9]	-0.51	0.17	-0.41	0.65	-0.60	-0.04	-0.26	0.21	0.03	0.41
16:1ω7%	0.67	-0.35	0.37	-0.75	0.79	0.08	0.36	-0.05	0.24	-0.10
18:1ω9%	-0.17	0.22	0.01	0.40	-0.20	-0.17	-0.12	-0.25	-0.54	-0.39
18:1ω7%	-0.05	0.08	0.23	0.44	-0.11	-0.16	0.03	-0.28	-0.34	-0.05
22:1ω9%	0.01	-0.12	0.12	0.41	-0.11	0.29	0.01	-0.15	-0.16	-0.23
∑MUFA%[10]	0.46	-0.25	0.45	-0.34	0.52	0.34	0.25	-0.29	-0.07	-0.37
16:3ω3%	0.56	-0.37	0.35	-0.77	0.79	0.12	0.36	0.03	0.36	0.04
16:4ω3%	-0.20	0.26	-0.31	0.18	-0.29	-0.23	-0.04	0.20	-0.08	-0.65
16:4ω1%	0.58	-0.39	0.31	-0.68	0.72	0.11	0.33	0.04	0.37	0.08
18:2ω6%	-0.17	0.27	-0.04	0.09	-0.15	-0.29	-0.18	-0.09	-0.37	-0.41
18:3ω3%	-0.16	0.51	-0.08	0.03	-0.01	-0.23	-0.15	-0.12	-0.50	-0.12
18:4ω3%	0.03	0.34	0.07	-0.06	0.07	-0.24	-0.03	-0.12	-0.27	-0.21
20:5ω3%	0.69	-0.25	0.34	-0.80	0.75	-0.06	0.35	-0.03	0.23	-0.17
22:5ω3%	-0.33	-0.07	-0.10	0.37	-0.44	0.12	-0.08	0.18	0.21	0.00
22:6ω3%	0.06	0.31	0.08	0.08	0.19	-0.29	-0.02	-0.22	-0.53	-0.15
∑PUFA%[11]	0.47	-0.05	0.29	-0.58	0.57	-0.13	0.21	-0.10	0.01	-0.39
PUFA/SFA	0.54	-0.11	0.33	-0.71	0.68	-0.07	0.28	-0.06	0.10	-0.39
∑ω3%[12]	0.39	0.08	0.26	-0.46	0.48	-0.24	0.18	-0.14	-0.09	-0.40
∑ω6%[13]	0.21	-0.17	0.19	-0.13	0.25	0.20	0.05	-0.07	-0.03	-0.35
DHA/EPA[14]	-0.30	0.29	0.04	0.40	-0.28	-0.10	-0.28	-0.37	-0.53	0.08
DHA% + EPA%	0.55	-0.06	0.29	-0.69	0.65	0.29	0.26	-0.11	-0.04	-0.21
Bacterial%[15]	0.23	-0.12	0.27	-0.24	0.33	-0.09	0.24	-0.09	0.06	-0.50
Diatom[16]	0.70	-0.35	0.32	-0.83	0.83	0.10	0.39	0.04	0.31	-0.11
Flagellate[17]	0.47	-0.39	0.16	-0.60	0.54	0.06	0.31	0.20	0.48	-0.14
Coastal margin%[18]	-0.19	0.46	-0.07	0.05	-0.08	-0.27	-0.18	-0.11	-0.50	-0.35
