# Supplementary material for: Phytoplankton fatty acid proportions in the Canadian Arctic are strongly affected by temperature, salinity, and phosphate in late summer
Source: PLoS One. 2026 Jan 22;21(1):e0340414. doi: 10.1371/journal.pone.0340414 (PMC12826509; doi:10.1371/journal.pone.0340414)
Supplement: S11 Table — Permutational analysis of variance (PERMANOVA) pairwise results among the nine OceanMet groups (shorthand area name) created from phytoplankton gathered from sub-surface chlorophyll maximum (SCM) waters from August 15 – October 4, 2021. Significance (p ≤ 0.05) between pairs is denoted by an asterisk next to the group. (PDF) [file pone.0340414.s019.pdf]

Groups	t	p
EHS/NWP/LS, DS-West/EBS	1.10	0.289
EHS/NWP/LS, DS/NWP	1.28	0.161
EHS/NWP/LS, BF/CAA*	2.09	0.002
EHS/NWP/LS, DS/DS-West*	1.72	0.013
DS-West/EBS, DS/NWP	1.19	0.222
DS-West/EBS, BF/CAA*	2.22	0.002
DS-West/EBS, DS/DS-West*	2.01	0.029
DS/NWP, BF/CAA*	2.57	0.001
DS/NWP, DS/DS-West	0.84	0.759
BF/CAA, DS/DS-West*	2.62	0.003
