# Supplementary material for: Phytoplankton fatty acid proportions in the Canadian Arctic are strongly affected by temperature, salinity, and phosphate in late summer
Source: PLoS One. 2026 Jan 22;21(1):e0340414. doi: 10.1371/journal.pone.0340414 (PMC12826509; doi:10.1371/journal.pone.0340414)
Supplement: Attachment — Submitted filename: Response to Reviewers_Revisions.pdf [file pone.0340414.s021.pdf]

Comments to Reviewers #1 and #2 are in italics throughout. Please note that the line numbers from the Reviewer may not now correspond to the revised manuscript. See the line numbers in the "Manuscript" (not the tracked changes) for the most up-to-date line location.

Reviewer #1: This study examines lipid classes, fatty acid markers, and phytoplankton assemblage metrics to evaluate oceanographic drivers of lipid content, composition, and quality across years in the Canadian Arctic. The dataset is valuable, and the study addresses important ecological questions regarding lipid synthesis and phytoplankton quality. However, the manuscript in its current form reads more like a draft than a publication-ready paper. The text is overly long, with the Results in particular providing more narrative than necessary. I recommend streamlining the manuscript by focusing on the key findings and using the figures to illustrate the results rather than explaining them in detail. I have provided specific comments and suggestions throughout that, if addressed, should strengthen the clarity and impact of the study.
Yes, thank you for your detailed recommendations, especially in regards to the Methods and Results. Both have been streamlined; we very much appreciate the effort Reviewer #1 took in making specific suggestions. We recognize that the submitted manuscript was quite descriptive and read as a narrative. The current version of the Results section should now be more concise and will allow the reader to focus more on the Conclusions and overall implications.

Line 38: Define DHA/EPA or note in parentheses that these are the marine omega 3 FAs
Agreed, DHA/EPA should be highlighted. Since these are not the only marine omega 3 FA of importance (there is also alpha-linolenic acid, 18:3ω3), we restated the phrase "two essential fatty acids" from the beginning sentence in the Abstract. This also allowed us to stay below the 300 word limit for the Abstract.

Line 72-86: Nice summary
Thank you!

Line 107: This should be North Water Polynya (not North Way Polynia). Also polynya is spelled incorrectly.
Agreed, this was changed throughout. We also decided to spell out all region names in the body of the manuscript (North Water Polynya vs NWP) since there are many acronyms that already pertain to fatty acids.

Line 119: condition may be fine alone, without "healthfulness". Or perhaps nutritional quality would be better
Agreed, "nutritional quality" is a more accurate descriptor (Line 124)

Line 122-123: Do you have a reference for this statement?
Yes, this was added.

Line 124 coastal or riverine/fluvial/terrestrial
Agreed, this was added (Line 132).

Line 139: Italicize Amundsen
Agreed, this was done throughout.

Line 141: Seawater for both filtered phytoplankton and nutrient analyses
Agreed, this was added.

Line 144-145: Change to "Filters were pre-combusted at 450 °C for 12 h and stored in aluminum foil."
Agreed, this sentence was changed (Line 155).

Line 146: Also -20 freezer at the lab?
Yes, this was added (Line 156).

Line 146-153: I'm confused by the statement and maybe just not understanding as written. Why were three filters combined for each station in 2021 and only one for 2019?
When collecting in 2019, we found that one filter was not enough for some stations and those stations were therefore not used in 2019. The stations used in our study were the ones that happened to have enough material for analyses. To eliminate this possibility, we used three filters in 2021 to ensure there was enough material for both lipid class and fatty acid analyses for all stations.
The collection was also time sensitive, so instead of filtering more water on one filter (which would clog up and inevitably slow down the filtering), multiple filters were used while keeping the amount of water filtered the same.
Clarifications were added to the paragraph to explain why different filter numbers were used for different years and/or areas (Lines 158-163).

Line 185: missing bracket/reference
Yes this was fixed.

Line 190-191: Awkward sentence. Revise to "The extracts were applied to silica gel–coated Chromarods using a micropipette."
Line 191-192: Perhaps scrap previous sentence and start with "Lipids were separated using a four-step development procedure on silica gel Chromarods."
Agreed, this was changed (Lines 203-204).

Line 191-198: Do you have a reference for this method that you followed or adapted?
Yes, this is referenced in Line 203.

Line 201: pre-combusted
Agreed, this was changed.

Line 201-202: volume of H2SO4-MeOH added?
Agreed, this was added (line 213).

Lines 213-217: Skewness not skewedness. Also note the tests used (examples in parentheses). Consider rephrasing to: "Twelve hydrographic and chemical variables (temperature, salinity, bottom depth, fluorescence, light transmission, oxygen saturation, dissolved oxygen, nitrogen probe, nitrate, silicate, phosphate, and ammonium) were evaluated for skewness (e.g. Shapiro–

Wilk test), homogeneity of variances (e.g. Levene's test), and collinearity (variance inflation factors, VIF) prior to analysis. Variables with skewed distributions were log-transformed. Concentration variables were also log-transformed to stabilize variances before statistical analyses."
Agreed, thank you very much. The suggested rephrasing is much more coherent (Lines 225-231).

Line 227: healthfulness health? As above, consider 'quality' or 'nutritional quality' instead?
Agreed, this was changed.

Line 229-231: Phrasing is a bit unclear. I would break them up rather than list 1-11. Ex: Metrics (of what?) used included total lipids, TAG/PL, TAG/ST. FA biomarkers included PUFA/SFA, Σω3, Σω6, and DHA/EPA. Phytoplankton assemblage markers were classified as bacterial, diatom, flagellate, or coastal margin.
Agreed, these sentences were changed (Lines 234-241).

Line 231-232: Explain why you do this. DHA and EPA are the marine-sourced omega-3 FAs. Did this differ much from the Σω3?
Yes, the Σω3 includes multiple other important fatty acids that can make up quite a large proportion (1-5%) in phytoplankton samples (alpha-linolenic and stearidonic for example). Furthermore, alpha-linolenic is considered essential. Many people only consider DHA and EPA, which is why we also put in DHA + EPA; however, we feel that these other phytoplankton sourced ω3 can be just as important in indicating the nutritional status of a phytoplankton assemblage. Proportionally, Σω3 was between 20-30% in most groups but DHA + EPA only made up between 9-20%. We think those ω3 other than DHA and EPA are probably more important than as historically been recognized.
A sentence was added to drive this point home (Lines 125-127).

Line 240: replace "is also discussed" to "were also assessed for…"
Agreed, this was changed.

Line 244 and 248, 249, 251: revise "oceanic" to "oceanographic"
Agreed, these were all changed

Line 256: "Arctic area" sounds strange since the entire study is in the Canadian Arctic. Suggest changing to "sites" or "stations"…. "although sometimes our resulting cluster groups were from the same stations."
Line 258: Same as above. Reconsider use of "Arctic areas"
Agreed, we changed "Arctic area" to region instead throughout the manuscript

Lines 212-279: The statistical analyses section is too dense and written as narrative. This section could be streamlined. I have tried to offer some suggested revised text below that incorporates some of my earlier comments:
"Twelve hydrographic and chemical variables (temperature, salinity, bottom depth, fluorescence, light transmission, oxygen saturation, dissolved oxygen, nitrogen probe, nitrate, silicate, phosphate, and ammonium) were evaluated for skewness (e.g. Shapiro–Wilk test), homogeneity of variances (e.g. Levene's test), and collinearity (variance inflation factors, VIF) prior to

analysis. Variables with skewed distributions were log-transformed. Concentration variables were also log-transformed to stabilize variances before statistical analyses.
Total lipids are reported in mg g^{-1} wet weight (WW), while lipid classes and fatty acids (FAs) are expressed as proportions (% total). Analyses were restricted to lipid classes and FAs contributing >1% of the total across both years, yielding eight lipid classes (of 14 possible) and 17 FAs (of 74 identified). In addition to individual groups, we used three lipid ratios (total lipids, TAG/PL, TAG/ST) and eight FA-based biomarkers (PUFA/SFA, Σω3, Σω6, DHA/EPA, bacterial, diatom, flagellate, coastal margin); DHA+EPA was also included for comparison despite overlap with Σω3.
Analyses were conducted separately for four groups defined by year and depth (2019 surface, 2019 SCM, 2021 surface, 2021 SCM). Correlation analyses were performed between all hydrographic variables and lipid classes, FAs, and biomarkers, and the number of significant correlations across groups was summarized.
Multivariate analyses were performed in PRIMER v7. Hierarchical cluster analyses with similarity profile tests (SIMPROF, Type 1, 999 permutations) were conducted for each year and depth using Euclidean distance. Outliers identified by SIMPROF were excluded. Final clusters, based on oceanographic properties only, were termed "OceanMet" groups; nutrient profiles were examined subsequently in relation to these groups. One-way PERMANOVA (Type III SS, 999 permutations) with Bray–Curtis similarity was used to test for differences among OceanMet groups, followed by pairwise tests. Principal coordinates analysis (PCO) was used to visualize relationships between OceanMet groups, lipid metrics, and biomarkers (TAG/ST excluded due to missing values), using log(x+1)-transformed data and Bray–Curtis similarity.
Finally, univariate comparisons of lipid classes and FAs among OceanMet groups were tested using one-way ANOVA with Tukey's HSD or, when assumptions of normality/variance were violated, non-parametric Kruskal–Wallis tests (Minitab v21)."
Agreed, this entire section was changed as suggested and all sentences were integrated (Lines 225-263). Again, thank you very much for writing a much more cohesive section; we appreciate the effort this must have taken.

Line 281–288: This section reads more like the methods/site description. Remove from results.
Agreed, this was removed.

Line 311: It is unnecessary to keep qualifying observations as "ocean" or "oceanic". This is known. Just say the inorganic nutrient measurements.
Agreed, these were changed to read "inorganic nutrient measurements" throughout.

Line 326-329: FAs have already been defined. Perhaps SFA, MUFA and PUFA should also be defined in the introduction and their relevance to quality indicators.
Agreed, if the FAs had been previously defined in the Introduction or Methods, abbreviations were removed from the Results section.

Results:
The Results section could be streamlined by using the figures to illustrate findings, rather than providing lengthy verbal descriptions of each panel. This would make the narrative more concise and easier to follow. Consider tightening the Results by highlighting the key patterns and directing the reader to the relevant figures, rather than describing each figure in detail.

Agreed, we removed any lengthy verbal descriptions that described more than one table, panel or figure.

Line 656: question mark in there that shouldn't be
Yes, this was removed.

Figures:
Line 391: Replace "mainly negative" with X out of X being negatively correlated. Suggest being more specific in the others too (Lines 402-405)
For the correlations, "mainly" was used because some correlations were positive and some were negative. However, if most of them were negative, but one was positive, then it would be incorrect to assume all were negative. If we were specific for each correlation, this section would not be as streamlined. For our dataset, when a correlation is "mainly" positive or negative with a suite of fatty acids, it's likely biologically relevant. Since many fatty acids have been found to be indicative of the same thing (i.e. 16:4ω1, 16:1ω7, and 20:5ω3 are all associated with diatoms), when many of them show the same pattern, or correlate, it's more meaningful than if one of those fatty acids had shown the pattern. Therefore, we have opted to keep the term "mainly" when describing the correlation patterns.

Lines 448-449: This is more discussion
Agreed, this was removed.

Line 470-472: Sentence is clunky and wordy. Suggest "Fluorescence was generally negatively correlated with coastal FA and biomarker signatures, and positively correlated with diatom-associated FAs and biomarkers."
Agreed, this was changed.

Lines 488-496: As before, this section should be incorporated in the methods/site description, or scrapped entirely. We don't need a descriptive account of the cruise tracks.
Agreed, this was removed.

Discussion:
Lines 732-742: Consider eliminating this paragraph. You don't need to tell us what you are about to tell us. Just tell us.
Agreed, this was removed.

Line 765: refer the reader back to figure (Fig 2B)
Agreed, this was added.

Line 810: same as above, point to figure
Agreed, this was added.

Lines 818-819 and Line 950: Don't DHA/EPA positively correlate with temperature in Fig 4? Or is DHA/EPA different from DHA + EPA?
Yes, DHA/EPA is different from DHA + EPA. DHA/EPA has been found to be a useful indicator of dinoflagellates (phytoplankton assemblage) whereas DHA + EPA has historically been used as

an indicator of nutritional quality. As we've stated above, we don't feel that DHA + EPA tells the whole story, which is why we use the overall Σω3 metric as well. Readers may want to know the DHA + EPA values and patterns (especially in regards to aquaculture), so that is one reason we decided to add DHA + EPA. The biomarker for flagellates is quite broad so adding in DHA/EPA is a way start to differentiate flagellate groups.
A sentence was added to differentiate the marker for DHA/EPA (Line 239).

Line 829: Reference style differs from the rest of the paper (numeric)
Agreed, this was changed.

Lines 850-851: Is that what you see in your dataset?
Not necessarily, but it's hard to say given that we don't have any spring data from these stations with which to compare. The comment below addresses this.

Lines 876-887: Why are we talking about spring blooms in September-October? It seems reasonable to me that dinoflagellates would have dominated the phytoplankton community at this time of year over diatoms. Was sample collection date assessed to have any statistical significance? It would also be interesting to have a metric related to sea ice break up date or some other indicator of length of time from spring bloom to sampling date.
We absolutely agree with you. What we found so interesting was that even though dinoflagellates and flagellates dominate the phytoplankton community over diatoms in the later summer/early fall, we still saw differences in fatty acids indicative of diatoms. So while we agree the absolute values of flagellates is likely higher during this time of year, we still saw evidence suggestive of high diatom abundance relative to other stations. However, we agree that we still were limited by successive sampling (as is most Arctic dataset sampling). We did not include sample collection date in the SIMPROF model since our question really only related to which oceanographic and nutrient metrics influenced fatty acid patterns. This dataset could very much be re-analyzed with length of time as a metric. For 2019 that would be interesting since it starts in mid-summer, but we expect that 2021 might actually control for length of time since sampling started in later in August and went through October. But for this manuscript, we believe our results can be discussed in relation to the seasonal phytoplankton patterns in the Arctic.

Line 913: Consider reviewing Ardynya and Arrigo (2022) review:
https://www.nature.com/articles/s41558-020-0905-y There is some information in here about relationships between nutrients and phytoplankton communities
Yes, we referred to this article quite a bit. What we found interesting is the precision with which the authors link major phytoplankton taxa and environmental drivers. Unfortunately, with fatty acids alone, this can't be done so it's hard to estimate how individual species will react given nutrient changes .Our data agrees with the authors conclusions regarding nitrogen availability, in that nutritional quality of phytoplankton is probably tightly linked to that particular nutrient, especially near those coastal, shelf break areas in Baffin Bay and the edge of the Beaufort Sea.

Line 924-928: My guess is that sample collection date is the important factor here.
Yes we agree. However, we still think it's important to consider that some stations up north and into September still showed results indicative of diatoms. It's worth further investigation to assess the phytoplankton assemblage that can then be paired with our fatty acid data.

Figures:
Figure 1: “polynia” should be polynya
Agreed, this was changed.

Figure 2: Reconsider the phrasing to describe the Tukey comparisons and letters used in the figure. I think I know what you mean here but it could be better explained in the figure caption. Consider combining Figs 2 and 5 and Figs 3 and 6 for ease of comparison between years.
Agreed, the captions for both Fig 2 and Fig 5 are reworded. We did consider combing the figures, but the goal with the SIMPROF groups is not to analyze interannual variability. Plus, the different groups between years make the resulting figure quite busy.

Figure 4: I like this figure a lot. Much clearer to understand the bigger picture. Caption refers to Supplemental Tables X-X?
Yes, thank you for catching that! The caption was changed.

Reviewer #2: Review:
Phytoplankton fatty acid proportions in the Canadian Arctic are strongly affected by temperature, salinity, and phosphate in late summer

Carlissa D. Salant, Jean-Éric Tremblay, Christopher C. Parrish

The authors have produced an impressive dataset on plankton lipid classes and fatty acid compositions from the Canadian Arctic, covering a wide range of stations, two different sampling years and two depth strata. The data were linked to a range of environmental predictors (e.g. temperature, light, nutrient concentrations) to understand the potential changes in nutritional quality at the base of the Arctic food web. Even though the data are highly valuable, and the aim of the study is well defined, the manuscript requires substantial improvement.
There are two major comments:
1. The manuscript shows strong overlap with the study by Marmillot et al. (2018), in terms of sampling region and the range of lipid and environmental data that have been sampled, and also the type of analysis that has been applied to illustrate the results. Therefore, it is surprising that the authors did 'almost' ignore the Marmillot study in the Introduction and Discussion, only citing it with other studies in a different context, instead of expanding on the similarity of this particular work. It is important that the authors clearly indicate how this new study compares and extends from Marmillot et al. in both the Introduction and the Discussion. I suggest that key data are directly compared in an additional plot, to either illustrate further interannual differences and/or regional consistency across multiple years of sampling. (see also Nielsen et al 2023, https://doi.org/10.1016/j.dsr2.2022.105247)
Yes, there is a strong overlap with Marmillot et al., (2020, 2024). There are a few main differences as to why we did not directly compare the two datasets. The year in both of those papers was for 2016 and there was no goal to look into interannual variability (although we agree with the Reviewer that this would be an interesting manuscript on its own since a few stations are similar). However the main reason for not directly comparing the datasets has to do with the goals/questions of each manuscript. Marmillot et al., (2020) was able to compare fatty acid profiles with phytoplankton assemblage, which is incredibly valuable. This was a key focus of the manuscript and something that we did not consider for our study.
More importantly, Marmillot et al., (2020, 2024) used their own criteria to delineate four different oceanographic regions; in our study, our oceanographic regions were the result of SIMPROF. We did not choose the oceanographic regions because our interest was in "groups" that were more similar. In other words, Marmillot et al., (2020, 2024) chose to study location; we decided to study simulated groups based on similar oceanographic metrics. Further, it would be difficult to decide which key data to directly compare. Depending the reader, interest could range from individual fatty acids to biomarkers, so the question would need to be specific. Overall, we agree with the Reviewer, but a comprehensive comparison deserves its own manuscript.

2. I rarely came across a manuscript that sticks so close to the actual data and does open up so little to wider considerations. This is to the extent that statistical results are already presented in the Abstract and the manuscript-specific term 'OceanMet groups' still occurs 6 times in the Conclusion. On Line 262, the authors promised 'Overall conclusions, however, are discussed in more broad terms', but I can see no evidence. Can we foresee if a warmer, ice-free Arctic will have more nutritious algae? And, if not, which additional information would we required? A

Conclusion is usually aimed at a wider audience that wants to find the key message without going through the nitty-gritty details of the study. Also, the end of the Discussion or Conclusion is a good place to get back to the study hypotheses and look at the bigger picture – e.g. the future health and biodiversity of the Arctic food web or potential impacts on food provision for local communities/fisheries. Do your study results give any potential insights towards such topics that could create a take-home message for people that are not explicitly interested in biomarker fatty acids?
Agreed, the submitted manuscript was very descriptive. We tried not to make too many expansive predictions given that fatty acids and lipids only equate to "energy" and it can be difficult, when not paired with phytoplankton assemblage data, to ascertain how phytoplankton profiles may look in the future. Given our attempt at highlighting which oceanographic metrics are most important, we agree that we need to add more context to our actual data. We have rewritten the Conclusion section and removed many of the OceanMet terms so as to make this section more applicable to a broader audience.

Other comments:
3. I'm not sure why oxygen concentrations or saturations have been included as a parameter that potentially drives the lipid- and fatty acid composition of the phytoplankton. In the manuscript, I read (Line 904): 'Dissolved oxygen in the marine environment can limit primary production since it is coupled to the biogeochemical cycle of both nitrogen and phosphorus'. As these nutrients are measured directly, I would not include oxygen concentrations or saturations here, except the authors can provide evidence that oxygen saturations are potentially too low or too high in the Canadian Arctic for the production of certain lipids (so a direct driver of their composition).
Agreed, oxygen concentrations and saturations don't drive lipid or fatty acid composition directly. As our data shows however, there are correlations between oxygen saturation and various fatty acids. Furthermore, we included oxygen concentrations and saturations for multiple reasons. Firstly, the goal was to use the SIMPROF model to create simulated groups that are most similar based on oceanographic metrics, irrespective of the any fatty acid or lipid input. Secondly, if primary production is limited there is a potential shift in lipid availability due to phytoplankton abundance and/or assemblage. Finally, neither of these metrics co-varied with the nitrogen probe values or phosphorus. We therefore felt is more appropriate to leave the oxygen values in the SIMPROF model.

4. The authors did not name the biomarker fatty acids for the groups they want to trace (Line 231): 8) bacterial, 9) diatom, 10) flagellate, and 11) coastal margin. Different studies use different fatty acids as biomarkers, e.g. 16:1(n-7) or 16:4(n-1) or 20:5(n-3) for diatoms. This has to be clarified on Line 231, and in the PCA figures. Moreover, it has to be stated which fatty acids have been used to trace vascular plants and green macroalgae (Line 853), to distinct between dinoflagellates and haptophytes (Line 870), and between dinoflagellates and flagellates (Line 883).
Agreed, the specific fatty acids for each biomarker was added (Lines 237-241). To our knowledge, there is no way to distinguish between dinoflagellates and haptophytes with fatty acids. DHA/EPA has been used as a dinoflagellate indicator, although the flagellate biomarker (C_{18}PUFA/C_{16}PUFA) encompasses dinoflagellates. Vascular plants and macroalgae were

differentiated by the "coastal margin" biomarker; the sentence in Line 853 was reworded to make this clear (Lines 774-778).

5. The statement (Line 79): 'In general, with an increase in temperature, there is an increase in TAG, SFA and MUFA proportions in phytoplankton [24–26].' needs clarification. If it refers to cultured algae with temperature as a sole driver, there is supporting evidence, but if it refers to field studies in the Arctic then the statement is wrong. In the Arctic, temperature effects are masked by the lack of nutrients and light (see Schmidt et al. 2024, DOI: 10.1111/gcb.17090). Thus, EPA and DHA proportions were lower in the colder Central Arctic Ocean than in the warmer Arctic shelf regions, and in the Bering Sea and North Bering- and Chukchi Sea the DHA proportions significantly increased with temperature (while EPA proportions did not change) (see Schmidt et al. 2024, Supplement Fig. S5). This matches observations in the present study (see Line 830 and 892) and needs to be cited in this context as it supports accumulating evidence that the temperature-to-PUFA relationship is in the Arctic regions more complex than suggested by Hixon and Arts (2016) based on global data sets.
Yes, we absolutely agree with the Reviewer. The relationship between temperature and PUFA (especially DHA) is becoming more complex. As the Reviewer noted, our study adds to the evidence that what we see in the field may not match what we see in laboratory studies. As suggested, we have added a clarification to Line 80, and added citations to Lines 813-819).

6. Some labels on the PCA plots (Fig. 3 and Fig. 6) are overlaying and therefore not readable.
Agreed, the overlays were adjusted if it was not possible to discern between biomarkers. Biomarker descriptions were also added to the captions

7. Some wording is imprecise, e.g. Line 88: 'Nitrogen can limit primary production….'It's the lack of nitrogen, I assume. Or Line 138: 'Phytoplankton samples were collected …' What you sample with a GF/C filter is particulate matter that can also include sediment, hetero- and mixotrophs. These are only examples, and I suggest you check the text carefully again for your wording.
Agreed, this was changed throughout.

8. There are several sentences with an unclear message, which might come from using AI. The following just gives a few examples, but I would suggest the whole text is checked by a native English-speaking human for clarity.
Line 721: energetics
This word was kept as it is a commonly used term in lipid ecology

Line 747: in regard for correlating with hydrographic properties
Agreed, this was reworded.

Line 751: be different based proximity
Agreed, this was reworded.

Line 755: Similarly, the 2019 SCM OceanMet group differences were due to a high proportion of the polar lipid class in the high temperature/low oxygen saturation, nitrogen, phosphate

OceanMet group (all Store Hellefiske Bank stations) and a low proportion in the high oxygen saturation/low salinity OceanMet group (East Barrow Strait and North Water Polynya stations).
Agreed, this sentence was reworded.

Line 767: total lipids was
Agreed, this was reworded.

Line 878: temperature phytoplankton
Line 878: the increase in the expanding
Agreed, this was reworded.

Line 895: Given the narrow range within the Arctic for both the surface and SCM waters
Agreed, this sentence was removed.

Line 902: health phytoplankton
Agreed, this was changed to "healthy"

Line 781: As a limiting nutrient in the Arctic related to the magnitude of phytoplankton blooms [45,74,75]
Agreed, this was reworded.
